# Similar behavioral but different endocrine responses to conspecific interactions in hand-raised wolves and dogs

**DOI:** 10.1016/j.isci.2023.105978

**Published:** 2023-01-14

**Authors:** Gwendolyn Wirobski, Friederike Range, Evelien A.M. Graat, Rupert Palme, Tobias Deschner, Sarah Marshall-Pescini

**Affiliations:** 1 Domestication Lab, Konrad-Lorenz-Institute for Ethology, University of Veterinary Medicine, Veterinaerplatz 1, 1210 Vienna, Austria; 2 Unit of Physiology, Pathophysiology and Experimental Endocrinology, Department of Biomedical Sciences, University of Veterinary Medicine, Veterinaerplatz 1, 1210 Vienna, Austria; 3 Institute of Cognitive Science, Comparative BioCognition, University of Osnabrück, Artilleriestrasse 34, 49076, Osnabrück, Germany

**Keywords:** Canine behavior, Canine physiology

## Abstract

Domestication has altered dogs’ conspecific social organization compared to their closest, non-domesticated relatives, gray wolves. Wolves live in packs whose survival depends on coordinated behavior, but dogs rely less on conspecifics, which predicts greater cohesiveness in wolf than dog packs. Endocrine correlates such as oxytocin and glucocorticoids modulate group cohesion resulting in species-specific differences in social interactions. We found that although wolves’ and dogs’ observable behavioral reactions to a territorial threat and separation from the pack were similar, hormonal responses differed. Wolves’ but not dogs’ oxytocin and glucocorticoid concentrations correlated positively with territorial behaviors and only wolves showed increased glucocorticoid concentrations after separation from their pack. Together, results suggest stronger emotional activation to threats to group integrity in wolves than dogs, in line with their socio-ecology.

## Highlights

• Domestication and social ecology have affected canid social organization
• Wolves had higher stress levels during separation from their pack than dogs
• Both reacted similarly to territorial threats but showed different hormonal patterns
• Oxytocin was associated with synchronized movement in wolves but not dogs

## Graphical abstract

**Figure:**
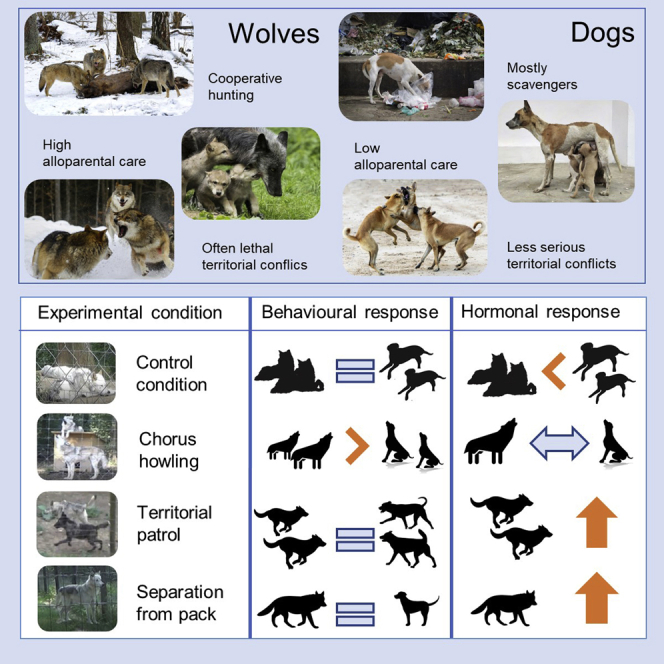

## Introduction

Gray wolves (*Canis lupus*) and domestic dogs (*Canis l. familiaris*) differ in important aspects of their social behavior and cognition (e.g., following human directions,^1^; conspecific cooperation,^2^; persistence in a problem-solving context,^3^). Thus far, these differences have largely been attributed to direct and indirect human selection for specific traits during the domestication process.^4^^,^^5^ The recently proposed Social Ecology (SE) hypothesis^6^ offers a more comprehensive perspective to compare wolf and dog behavior and cognition, taking into account species-specific ecologies.

Wolves are highly social and live in tightly knit packs formed of closely related,^7^ but also unrelated members.^8^ Members of a wolf pack coordinate their behavior and cooperate with each other extensively.^6^ They hunt cooperatively,^9^^,^^10^ share parental care of their offspring,^11^ and defend their territory together.^12^ Inter-pack competition is fierce and, in some wolf populations, the main cause of mortality.^12^^,^^13^ Intra-pack cohesion thus is essential to survival and can be reinforced by affiliative interactions (e.g., ‘conciliatory’ post-conflict interactions^14^). Furthermore, long-range vocalizations such as howling facilitate reunion with temporarily dispersed pack members,^15^^,^^16^^,^^17^ help to coordinate and synchronize group movements,^18^ and serve as a form of territorial spacing to avoid confrontations with rivaling packs.^19^^,^^20^

In contrast to wolves, the social ecology of free-ranging dogs (FRD) appears to rely less on pack cohesion for survival. FRD may live solitarily but also form dyads or packs,^21^ with food availability and reproductive season affecting group size and composition in some populations.^22^^,^^23^ FRD have a promiscuous mating system where females mate with multiple males – in contrast to strict suppression of subordinate breeding in wolves^24^ – lacking the cooperative care seen in wolf packs, although some incidences of alloparental behavior have been reported.^25^ FRD primarily scavenge on readily available human waste,^26^ which does not require coordinated group activity nor a particular tolerance of conspecifics as food items are often scattered and easy to obtain by one individual on its own.^23^ FRD packs engage in joint territory defense,^27^ with most inter-pack conflicts occurring at feeding and mating sites, and territorial boundaries.^28^ Interestingly, 80% of agonistic behaviors recorded during those encounters were low-level forms of aggression, such as ‘barking, growling, and snarling’ and ‘ritualized vocal duels without actual physical contact’,^28^ suggesting that inter-pack conflicts may be less severe in FRD than wolves. Taken together, it appears that studies of wolf-dog behavioral and cognitive differences would benefit from considering their social ecologies which – in addition to selection by humans – helped shape species-specific traits of modern-day wolves and dogs.^6^

This notion is supported by studies on captive animals: Wolves outperformed dogs in two tasks requiring attentiveness toward and coordination with conspecifics: a problem-solving task requiring imitation of a conspecific’s behavior^29^ and a ‘cooperative’ string-pulling task.^2^ Wolves were also more tolerant than dogs around food^30^ and showed prosocial behavior toward their pack mates in a touch screen task.^31^ However, all these studies were conducted in a feeding context. This leaves open the possibility that, although wolves appear more tolerant and cooperative with conspecifics than dogs in a feeding situation (potentially because of their reliance on cooperative hunting), in other contexts, such as territorial defense, pack members may be as important for dogs as for wolves. Hence, the present study aimed to investigate how the domestication process affected dogs’ coordination with conspecifics in a non-feeding context (i.e., behavior during territorial defense) and pack cohesion (i.e., response to forced separation from pack members and elicited chorus howling). In addition to recording behavior, we non-invasively collected urine samples to measure hormone metabolites because this potentially opens a window into the psycho-emotional state of the animals.^32^ Specifically, we focused on two well-studied endocrine markers that have previously been implicated in the social behavior and domestication process of dogs: Oxytocin (OT) and glucocorticoids (GCs).^33^^,^^34^^,^^35^^,^^36^^,^^37^^,^^38^

OT is a neuropeptide hormone released in response to pleasant interactions which promotes further contact with a social partner.^39^ In humans, OT release has been linked to group activities involving coordinated actions, such as chorus singing^40^^,^^41^^,^^42^^,^^43^ (but see^44^^,^^45^), synchronized movement,^46^^,^^47^^,^^48^^,^^49^ and behavioral coordination.^50^ Furthermore, elevated OT concentrations have been associated with helping in- but not out-group members,^51^ linking OT release to human parochial altruism and in-group conformity.^52^^,^^53^ In animals, OT is released during affiliative human-animal (e.g., in dogs^34^^,^^54^) but also conspecific interactions (e.g., grooming in chimpanzees^55^), and facilitates group activities during which individuals synchronize their actions (e.g., cooperative pup feeding and guarding in meerkats^56^). Of interest, OT concentrations were higher following group hunting and intergroup conflict in wild chimpanzees than in control situations with and without affiliation, demonstrating that OT release is strongly connected to coordinated actions with known individuals.^57^ Accordingly, OT seems to specifically promote in-group cooperation, which ultimately benefits group survival.

Glucocorticoids (GCs), such as cortisol and corticosterone, are released in high-arousal and stressful situations as a result of hypothalamo-pituitary-adrenal (HPA) axis activation. Besides their roles in energy and glucose metabolism, they also mediate social behavior.^58^ Elevated GC concentrations have consistently been linked to competitive, threatening, and potentially harmful events such as encounters with rivalling groups,^59^ social instability,^60^ and social defeat.^61^ Furthermore, GC secretion is associated with bond fragmentation and separation distress.^62^ In captive wolves, taking an individual away from its pack members was associated with increased howling and elevated GC concentrations in the remaining animals,^17^ and pack instability, particularly after the loss of dominant individuals, was associated with high fecal GC metabolite concentrations in wild wolf packs.^63^ Both findings indicate that separation and pack fragmentation produce a considerable stress response in wolves. In contrast, dogs separated from conspecific companions did not have elevated GC concentrations.^64^ In a more recent, comparative study, enclosure-living wolves showed more stress-related behaviors and escape attempts than dogs when briefly separated from their pack, but both species had increased salivary GC concentrations afterward.^65^ However, this study also included a variety of other events (e.g., presentation of a novel object); hence, reported changes in GC concentrations likely reflect the cumulative effect of these events and not only the response to separation.

Both OT and GCs have been implicated in the domestication process of animals. Most prominently, artificial selection experiments with silver foxes (a variant of the red fox, *Vulpes vulpes*) have demonstrated that selection for tameness rapidly led to lower HPA axis activity and thus lower circulating GC concentrations in tame compared to control foxes.^66^ Further work comparing domesticated to wild-type species has shown a similar pattern (guinea pigs, *Cavia aperea f. porcellus*, and cavies, *C. aperea*,^67^; chicken, *Gallus gallus domesticus*, and red jungle fowl, *Gallus gallus*,^68^), however others have found contrasting results (dogs, *C. lupus familiaris,* and wolves, *C. lupus*,^35^^,^^69^). OT’s potential role in animal domestication has primarily been discussed for dogs,^36^^,^^37^^,^^38^ but only very few empirical studies have been conducted to date, comparing wolves’ and dogs’ oxytocinergic response to human contact.^34^^,^^70^ Considerably more research was published on pet dogs’ and owners’ oxytocinergic reactivity to affiliative interactions (for a review, see^71^). Overall, the available data on OT and (pet) dogs suggests, that positive human-dog interactions lead to increased OT concentrations in both humans and dogs, however, given null findings (see for example^72^), this appears to depend on other factors as well.

In the present study, we tested comparably raised, group-housed wolves and dogs in three different conditions: (1) A mock territorial patrol to investigate differences in territorial and synchronized behavior and associated hormonal changes, (2) a forced separation from the pack members to evaluate behavioral and hormonal parameters of separation, and (3) an induced chorus howling event to measure differences in group vocalisations and underlying hormonal correlates. We note that although chorus howling (defined as two or more individuals howling together) has primarily been studied in wolves, howling is part of the vocal repertoire of all canid species, including domestic dogs^73^^,^^74^ and, in our study population, occurs in both wolf and dog packs. Furthermore, we collected urine samples from each animal following an undisturbed period in the home enclosure with their pack mates present (control condition). To control for varying levels of activity between the control and test conditions, we included the normalized duration of locomotor activity as a control variable in all hormonal models.

**Figure 1:**
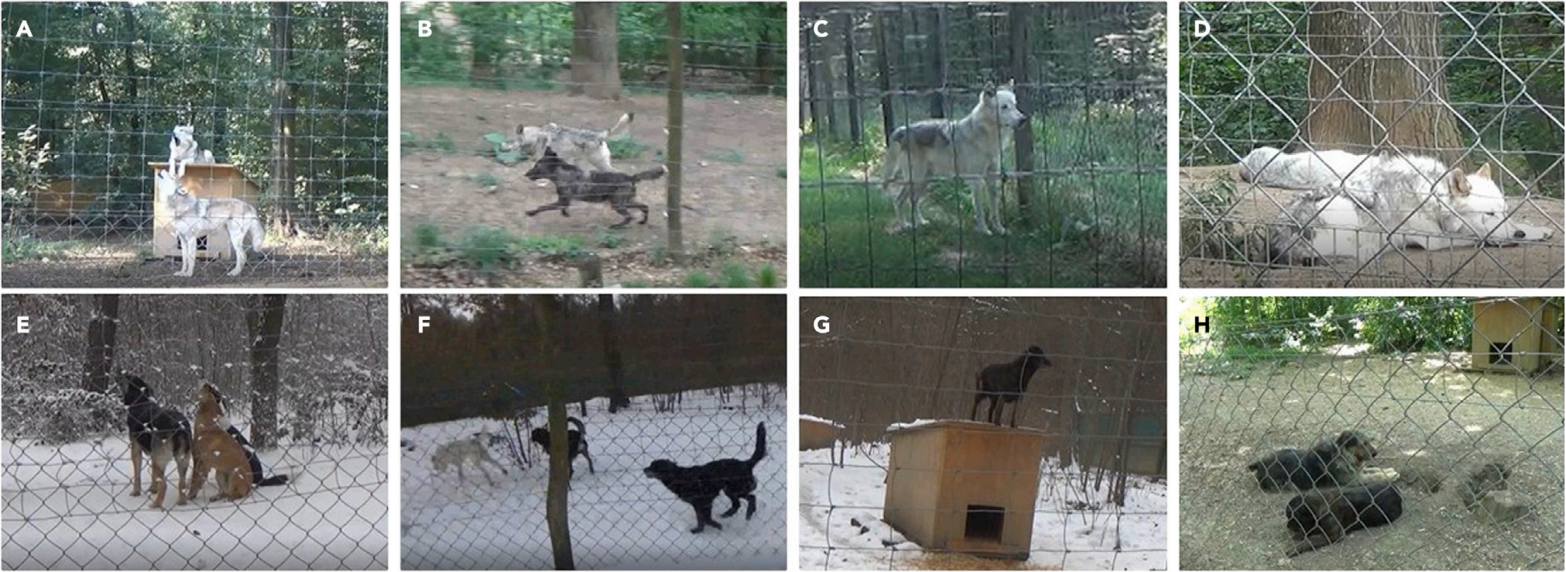
Overview of conditions (A–H) Experimental and control conditions in wolves and dogs. Induced chorus howling (A: wolves, E: dogs); mock territorial patrol (B: wolves F: dogs); separation from pack (C: wolf; G: dog); undisturbed period in familiar environment with pack mates present/control condition (D: wolves, H: dogs).

Based on the different socio-ecologies of the two species, and wolves’ higher dependence on pack members than dogs,^6^ we hypothesized that pack cohesion during territorial patrols and chorus howling, as well as separation distress during isolation from pack members would be more evident, from both a behavioral (see Table 1 for the ethogram and Figure 1 for an overview of conditions) and hormonal perspective, in wolves than dogs. Specifically, we predicted (1) more synchronized and territorial behavior, as well as higher urinary OT metabolite (uOTM) concentrations following induced chorus howling and territorial conditions (relative to the control condition), in wolves than dogs; (2) more escape attempts, reunion-promoting vocalizations (such as solo howling), and higher urinary GC metabolite (uGCM) concentrations following the separation from the pack (relative to the control condition) in wolves than dogs. We further predicted that, in both wolves and dogs, (3) uOTM concentrations would be associated positively with synchronized and territorial behavior, as well as chorus howling, and that higher uGCM concentrations would be linked to more escape- and stress-related behaviors during separation, as well as more territorial behavior. In addition, we investigated the association between uOTM and uGCM concentrations, in a species- and context-specific manner, because oxytocinergic activity is known to modulate HPA axis activity and the release of GCs, depending on social context (stress buffering effect of social contact^75^^,^^76^^,^^77^^,^^78^^,^^79^). Previously, we reported a positive correlation between unstimulated uOTM and uGCM concentrations in wolves but not dogs.^35^ Based on this finding and the stress buffering effect of OT and wolves’ greater reliance on conspecific partners, we predicted (4) a stronger positive correlation of uOTM and uGCM concentrations in wolves than dogs, particularly in conditions where pack mates were present (control condition, mock territorial patrol, chorus howling), but not following separation. However, given the paucity of data on the time course of urinary OT and GC secretion in our study species, alternatively, a negative or no correlation between uOTM and uGCM concentrations may be found.

**Table 1:** Ethogram of coded behaviors used for statistical analyses

Category	Behavior	Description
**Activity state**	Locomotion	Moving around in enclosure, either running or walking
	Resting	Lying down in enclosure, eyes may be closed or open
	Immobile	Sitting down or standing on all four legs, not moving
	Out of sight	Animal not visible. Coded when the whole body is out of sight, or when it is impossible to see on the video what the animal is doing.
**Simultaneous howling**	Chorus howl	The animal elicits a high-pitched prolonged sound, often the head is held up and back (snout in the air, ears back, tail may be tucked under); one or more other individuals from same pack join in, resulting in a chorus
**Solo vocalizations**	Solo howl	The animal elicits a high-pitched prolonged sound, often the head is held up and back (snout in the air, ears back, tail may be tucked under), no other individual joins the howler
	Whine	A high-pitched sound, sometimes precedes a howling bout but may also occur separately
**Territorial behaviors**	Simultaneous marking	Urinating, defecating and/or ground scratching in proximity (within 3 body lengths) and within 10 s of a pack member, or urinating/defecating on the same spot as a pack member within 10 s of each other
	Ground scratch	To scratch the ground with front and/or hind legs, often following defecating or urinating. May be used as a threat display, sometimes facing the neighboring pack.
	Patrolling along fence	Moving along the fence perimeter, usually circling around in the enclosure, tail held high and/or hackles raised, stopping from time to time to watch the neighboring pack
**Escape/distress-related behaviors**	Pacing	Continuous movement without apparent aim, from left to right in a straight line placing the feet exactly in the same position each way, may appear to be looking for a way out, tail usually held low
	Bite fence	Biting into the enclosure fence
	Dig fence	Digging into ground, usually at the fence or next to the shifting gate
**Synchronized behaviors**	Synchronous locomotion	Moving around in the enclosure in proximity to each other (within 3 body lengths)
	Synchronous resting	Lying down in proximity to each other (within 3 body lengths)
	Synchronous immobile	Standing or sitting in proximity to each other (within 3 body lengths), one may be lying down but both animals must be immobile, often while watching something
**Affiliative behaviors**	Social sniff	Sniffing each other
	Grooming	Licking or nibbling each other
	Resting in body contact	Resting or sleeping in physical contact with another pack member
**Agonistic behaviors**	Threat	Growling and often baring of the teeth toward another pack member
	Fight	High intensity, aggressive, often damaging encounter
	Chase off	Chasing away a pack member, usually a threat is displayed before

Behaviors were recorded as durations and subsequently normalized for the total time the animal was visible on the video.

## Results

### Hormonal and behavioral correlates of pack cohesion

#### Urinary oxytocin metabolites

To investigate whether wolves and dogs differed in their uOTM concentrations according to the test conditions, a model was fitted including feeding status, reproductive phase, locomotion, and the interaction between species and sex as control variables, as well as random effects of subject, pack, and assay plate.

The interaction effect of species and condition on uOTM concentrations was not significant (LRT = 4.0, df = 3, p = 0.261; Table S1, supplemental information) and neither was the main effect of condition (LRT = 7.2, df = 3, p = 0.066; Figure S1, supplemental information, Table S1, supplemental information), but dogs had higher uOTM concentrations than wolves regardless of condition (LRT = 8.7, df = 1, p = 0.003; Table S1, supplemental information). Males of both species had higher uOTM concentrations than females (LRT = 4.9, df = 1, p = 0.027; Table S1, supplemental information). In wolves, fasted individuals had higher uOTM concentrations than fed ones (LRT = 14.1, df = 1, p = 0.000; Table S1, supplemental information).

#### Synchronized behavior

To test whether wolves and dogs showed different patterns of synchronous behaviors and whether those were linked to uOTM concentrations, three models were fitted. The first model used all synchronous behaviors as the response variable and included species interacting with condition as the test predictor, sex as a control variable, and subject and pack as random effects. The second model was fitted in the same way but using only synchronized movement as the response. The last model explored whether there was a species-specific effect of synchronized movement on uOTM concentrations, while accounting for sex, feeding status, and reproductive phase. Subject, pack, and plate were included as random effects.

**Figure 2:**
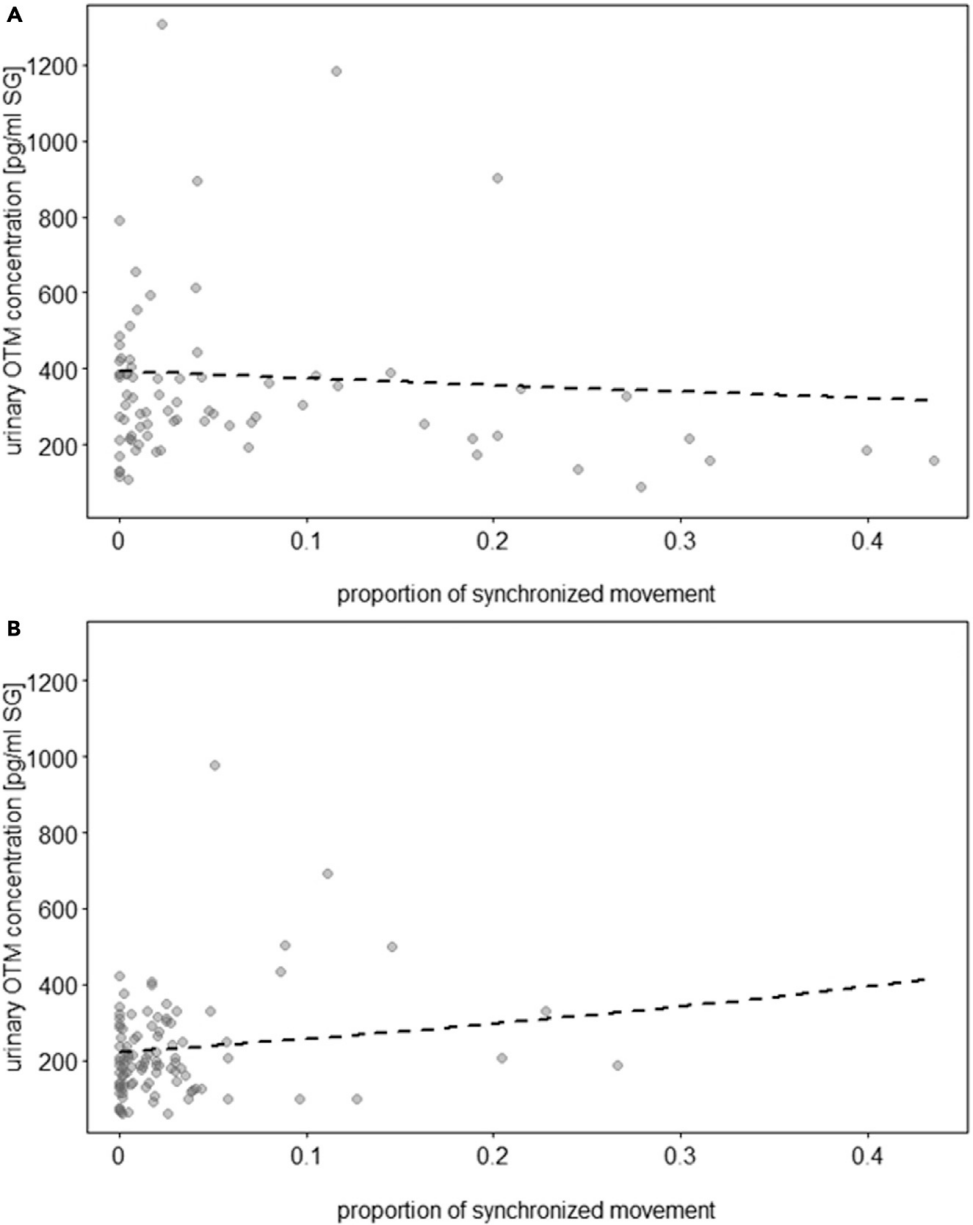
Oxytocin and synchronized movement (A and B) Association of synchronized locomotion and urinary OTM concentrations (pg/mL, corrected for specific gravity, SG) in a) dogs (N = 10 individuals) and b) wolves (N = 9 individuals). The dashed lines represent the fitted model for the effect of synchronized locomotion on urinary OTM concentrations, given all control predictors and random factors. Each dot represents a sample. Each animal provided 1–3 samples (the model accounted for repeated sampling of the same individuals).

Species and condition were not significantly associated with synchronous behaviors (synchronized resting, immobile, and locomotion grouped together) (LRT = 3.3, df = 5, p = 0.661). However, when synchronized movement was analyzed separately, dogs showed more synchronous locomotion in the territorial but less in the chorus howling condition than wolves (LRT = 11.9, df = 2, p = 0.003; Table S2, supplemental information). Furthermore, synchronized locomotion was positively associated with uOTM concentrations in wolves but not dogs (LRT = 3.9, df = 1, p = 0.049; Table S3, supplemental information; Figure 2).

#### Chorus howling

Similarly to the approach described above for synchronized behavior, three models were fitted; the first was designed to test for a species- and condition-specific difference in the proportion of chorus howling, the second to investigate the link between induced chorus howling and uOTM, and the third was fitted specifically to test the effect of spontaneous chorus howling on uOTM (all models including the same control variables and random effect structure as described above for synchronized behavior and in the STAR Methods section).

The interaction between species and condition (induced chorus howling versus control condition) was significant (LRT = 8.1, df = 1, p = 0.004; Table S4, supplemental information). Specifically, the wolves chorus howled significantly longer in response to the siren than the dogs and compared to the control condition, but there was no association between the duration of induced chorus howling and uOTM concentrations (LRT = 0.52, df = 1, p = 0.473) in either species. Furthermore, spontaneous chorus howling was not significantly associated with uOTM concentrations (LRT = 0.34, df = 1, p = 0.569).

#### Territorial behavior

Two models were fitted; the first investigated whether wolves and dogs showed differences in territorial behavior according to condition, and the second tested for an effect of territorial behavior on uOTM concentrations (both models including the same control variables and random effects as described for synchronized behavior and in the STAR Methods section).

**Figure 3:**
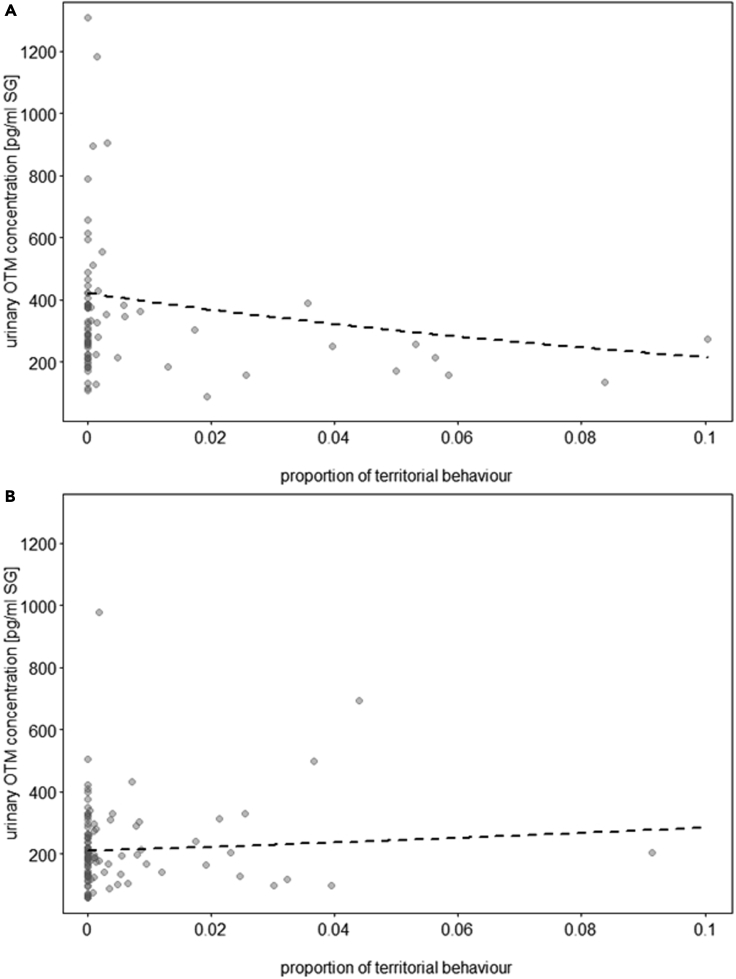
Oxytocin and territorial behavior (A and B) Association of territorial behavior and urinary OTM concentrations (pg/mL, corrected for specific gravity, SG) in (A) dogs (N = 10 individuals) and (B) wolves (N = 9 individuals). The dashed lines represent the fitted model for the effect of territorial behavior on urinary OTM concentrations, given all control predictors and random factors. Each dot represents a sample. Each animal provided 1–3 samples (the model accounted for repeated sampling of the same individuals).

Species was not a significant predictor of territorial behavior (ground scratching, patrolling along fence, simultaneous marking) (LRT = 0.3, df = 1, p = 0.567; Table S5, supplemental information), but dogs and wolves showed significantly more territorial behavior during the mock territorial patrol than the control condition (LRT = 35.8, df = 1, p = 0.000; Table S5, supplemental information). Territorial behavior was related positively to uOTM concentrations in wolves but not dogs (LRT = 5.3, df = 1, p = 0.021) (Figure 3A–B, Table S6, supplemental information).

#### Affiliative behavior

Two models were fitted; the first investigated whether wolves and dogs showed differences in affiliative behavior according to condition, and the second tested for an effect of affiliative behavior on uOTM concentrations (both models including the same control variables and random effects as described for synchronized behavior and in the STAR Methods section).

Species and condition were not significantly associated with affiliative behavior (LRT = 1.9, df = 7, p = 0.967), nor did affiliative behavior affect uOTM concentrations in either wolves or dogs (LRT = 1.2, df = 1, p = 0.281).

### Hormonal and behavioral correlates of territorial defense and separation

#### Urinary glucocorticoid metabolites

To investigate whether wolves and dogs differed in their uGCM concentrations according to the test conditions, a model was fitted including feeding status, reproductive phase, locomotion, and the interaction between species and sex as control variables, as well as random effects of subject, pack, and assay plate.

**Figure 4:**
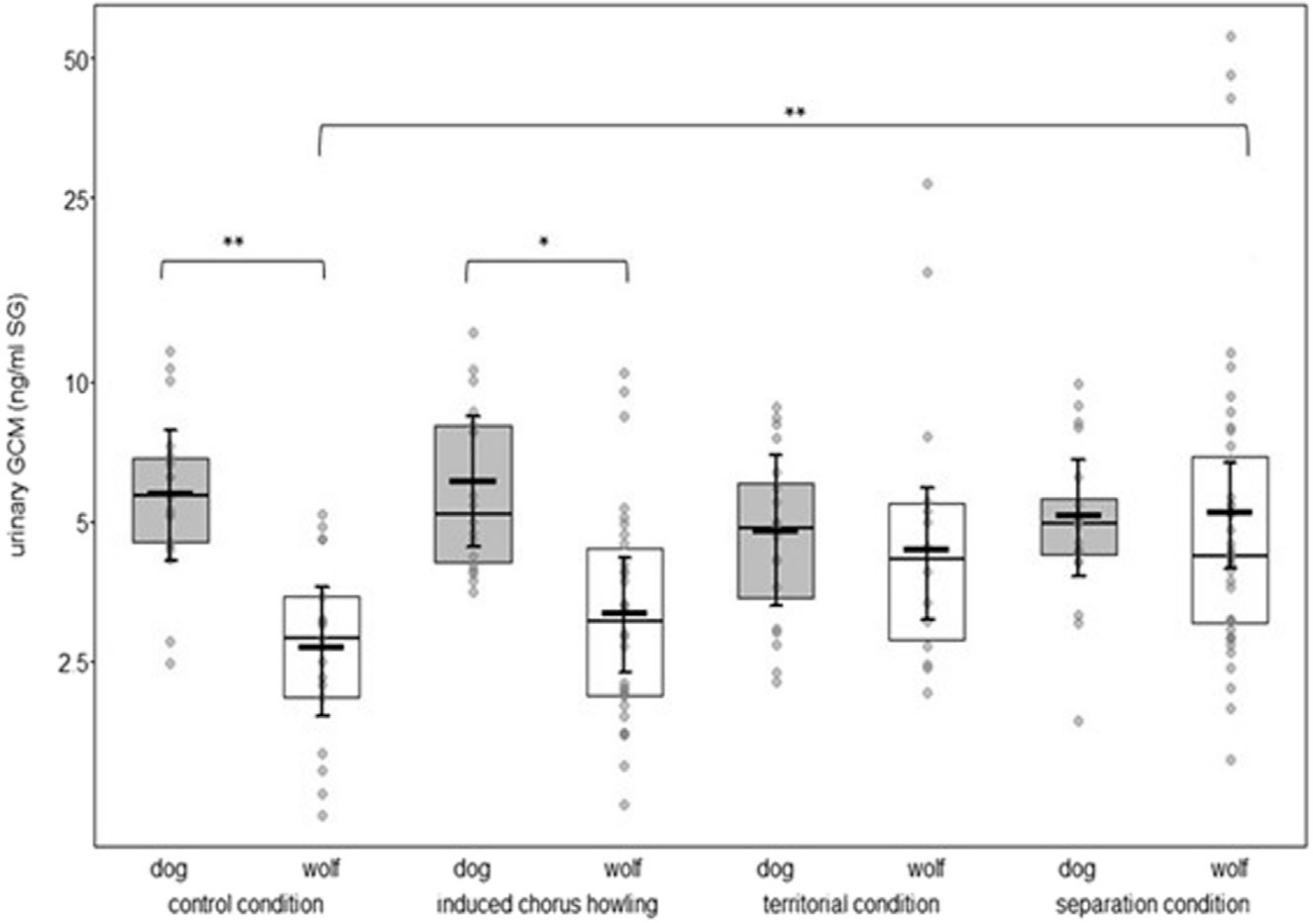
Glucocorticoids across conditions Urinary GCM concentrations (ng/mL SG) of wolves (N = 10; white boxes) and dogs (N = 10; light gray boxes) across test conditions Indicated are medians and quartiles (horizontal lines with boxes) as well as the fitted model and its 95% confidence intervals (thick horizontal lines with error bars). Gray dots represent individual samples. ∗∗p ≤ 0.01, ∗p ≤ 0.05.

The interaction between species and condition (LRT = 16.6, df = 3, p = 0.001; Figure 4, Table S7, supplemental information) was significant. Dogs had higher uGCM concentrations than wolves following the control (p = 0.009, Table S8, supplemental information) and induced chorus howling (p = 0.025, Table S8, supplemental information) conditions. There was no effect of condition on uGCM concentrations in dogs, but wolves had significantly elevated uGCM concentrations following the separation condition relative to the control and induced chorus howling conditions (p = 0.004 and p = 0.045, respectively; Table S9).

#### Territorial behavior

To test for an effect of territorial behavior on uGCM concentrations, the model was fitted in accordance with the one described above to test for an effect on uOTM concentrations.

**Figure 5:**
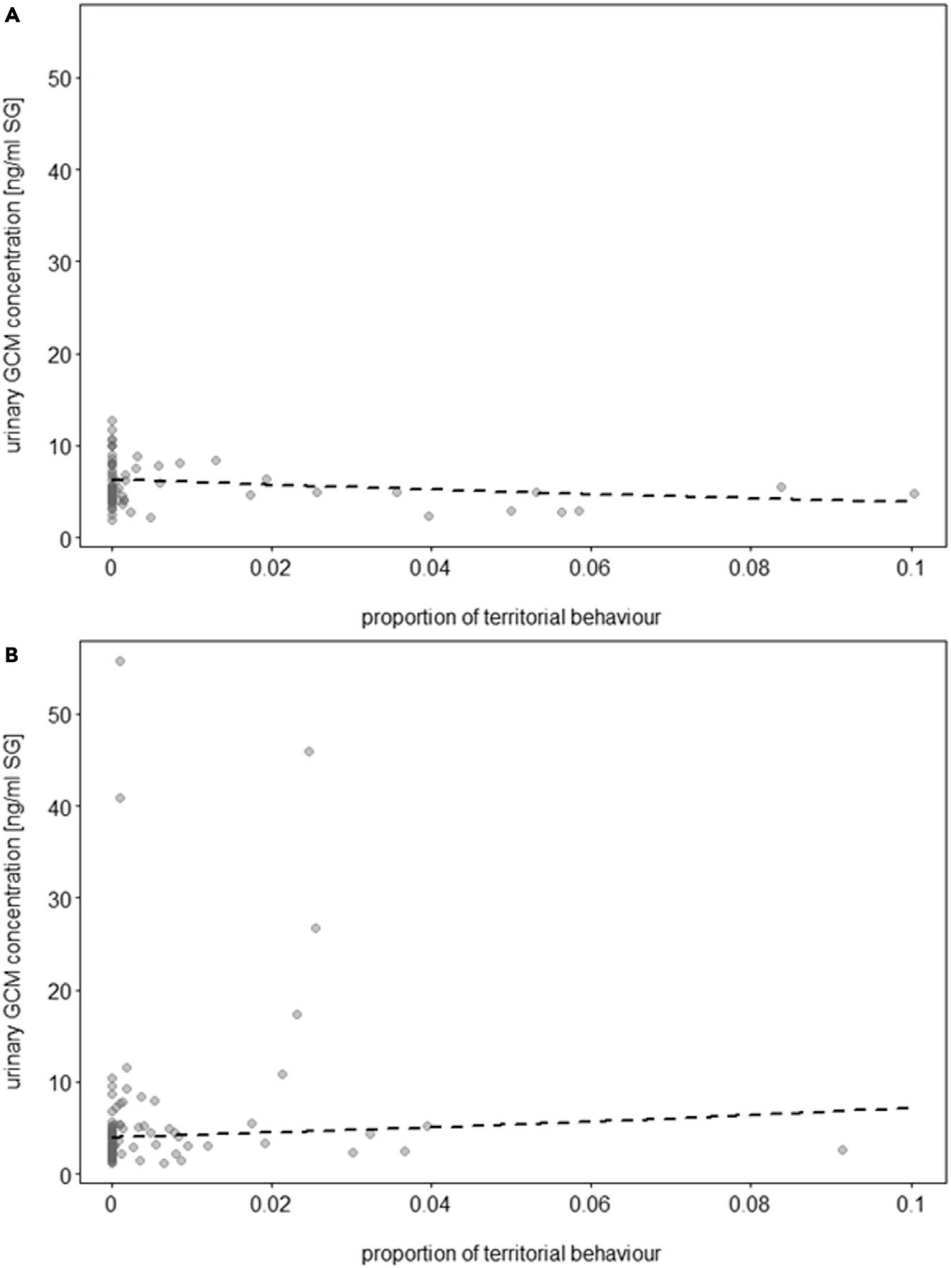
Glucocorticoids and territorial behavior (A and B) Association of territorial behavior and urinary GCM concentrations (ng/mL, corrected for specific gravity, SG) in a) dogs (N = 10 individuals) and b) wolves (N = 9 individuals). The dashed lines represent the fitted model for the effect of territorial behavior on urinary GCM concentrations, given all control predictors and random factors. Each dot represents a sample. Each animal provided 1–3 samples (the model accounted for repeated sampling of the same individuals).

Territorial behavior was positively linked to uGCM concentrations in wolves but not dogs (LRT = 4.3, df = 1, p = 0.038; Figure 5A–B, Table S10, supplemental information).

#### Escape-related behavior

Two models were fitted; the first investigated whether wolves and dogs showed differences in escape-related behavior according to condition, and the second tested for an effect on uGCM concentrations (both models including the same control variables and random effects as described for synchronized behavior and in the STAR Methods section).

There were no significant differences between wolves and dogs or conditions (separation versus control condition) in the occurrence of escape behaviors (pacing, biting/digging at enclosure fence) (LRT = 2.7, df = 3, p = 0.447). However, three wolves showed markedly more escape attempts during separation from their pack than the other individuals. There was a positive association between escape-related behavior and uGCM concentrations in both species (LRT = 6.2, df = 1, p = 0.013; Figure S2, Table S10, supplemental information).

#### Solo howling

Two models were fitted; the first investigated whether wolves and dogs showed differences in solo howling according to condition, and the second tested for an effect on uGCM concentrations (both models including the same control variables and random effects as described for synchronized behavior and in the STAR Methods section).

**Figure 6:**
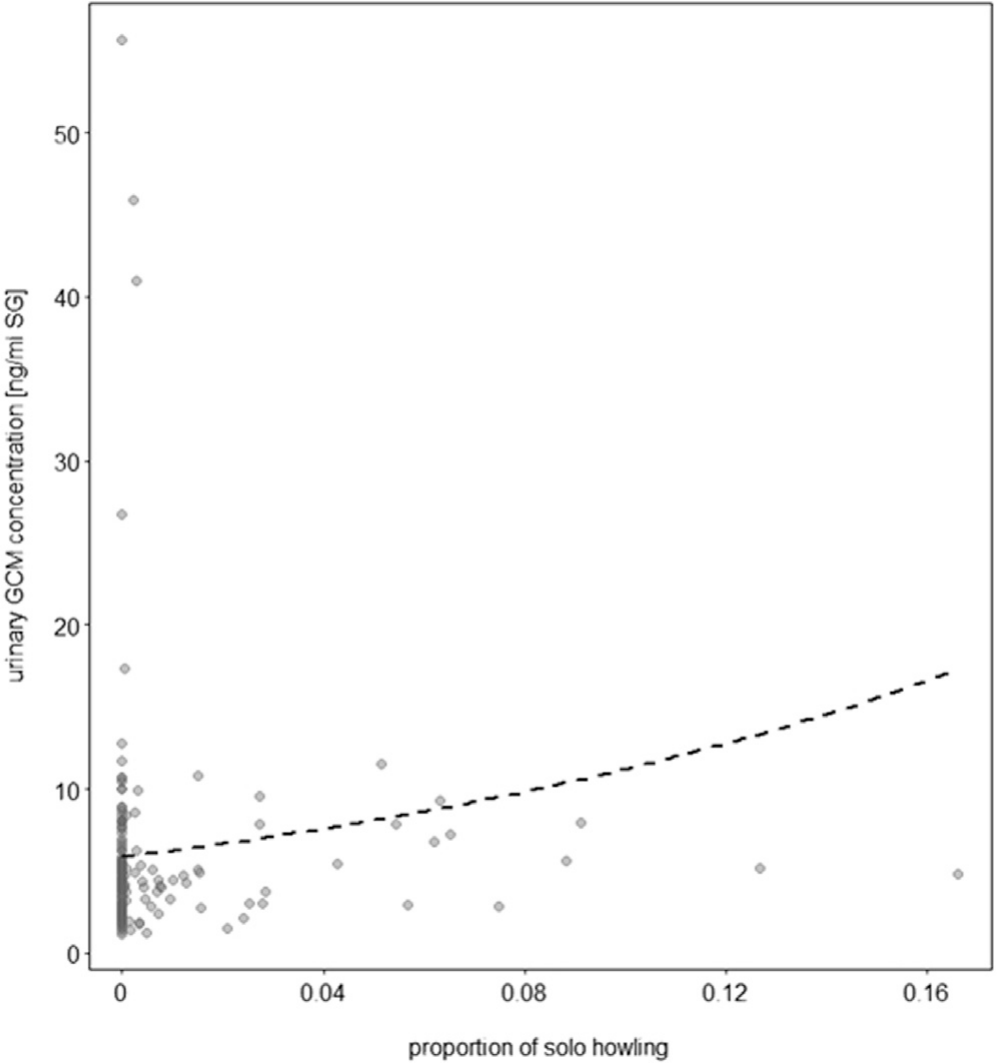
Glucocorticoids and solo howling Association of solo howling and urinary GCM concentrations (ng/mL, corrected for specific gravity, SG) The dashed lines represent the fitted model for the effect of solo howling on urinary GCM concentrations, given all control predictors and random factors. Each dot represents a sample. Each animal provided 1–3 samples (the model accounted for repeated sampling of the same individuals).

There were significant main effects of both species and condition (separation versus control condition) on solo howling: Wolves solo howled longer than dogs (LRT = 5.5, df = 1, p = 0.019; Table S11, supplemental information) and both howled longer during the separation from their pack members than during the control condition (LRT = 7.5, df = 1, p = 0.006; Table S11, supplemental information). There were positive associations between solo howling and uGCM concentrations in both species (LRT = 11.8, df = 1, p = 0.001; Figure 6, Table S10, supplemental information).

#### Whining

Two models were fitted; the first investigated whether wolves and dogs showed differences in whining according to condition, and the second tested for an effect on uGCM concentrations (both models including the same control variables and random effects as described for synchronized behavior and in the STAR Methods section).

Dogs whined significantly more than wolves when separated from their pack members and both whined more in the separation compared to the control condition (LRT = 18.9, df = 1, p = 0.000; Table S12, supplemental information). Whining and uGCM concentrations were not linked (LRT = 0.4, df = 1, p = 0.534; Table S10, supplemental information).

#### Locomotion

Three models were fitted; the first investigated whether wolves and dogs showed differences in locomotor activity according to condition, and the second and third tested for an effect on uOTM and uGCM concentrations (both models including the same control variables and random effects as described for synchronized behavior and in the STAR Methods section).

Wolves moved around significantly more than dogs during the separation condition, but less during the induced chorus howling and territorial conditions (LRT = 15.1, df = 3, p = 0.002; Table S13, supplemental information). Both wolves and dogs moved more during the territorial than during the control condition, but the proportion of locomotion did not significantly affect uOTM (LRT = 1.7, df = 1, p = 0.188; Table S1) or uGCM (LRT = 0.1, df = 1, p = 0.806; Table S10, supplemental information) concentrations.

#### Agonistic behavior

Two models were fitted; the first investigated whether wolves and dogs showed differences in agonistic behavior according to condition, and the second tested for an effect of agonistic behavior on uGCM concentrations (both models including the same control variables and random effects as described for synchronized behavior and in the STAR Methods section).

Species and condition were not significantly associated with agonistic behavior (LRT = 8.2, df = 7, p = 0.311), nor did agonistic behavior affect uGCM concentrations in either wolves or dogs (LRT = 0.0, df = 1, p = 0.856) (it should be noted that intra-pack agonistic behavior was observed only 10 times during 182 observations in total).

### Link between oxytocin and glucocorticoid concentrations

To investigate the species- and condition-specific link between uOTM and uGCM concentrations, we fitted four models (using only the data from one condition for each) with uGCM as the response variable. Random effects of subject, pack, and plate were included.

**Figure 7:**
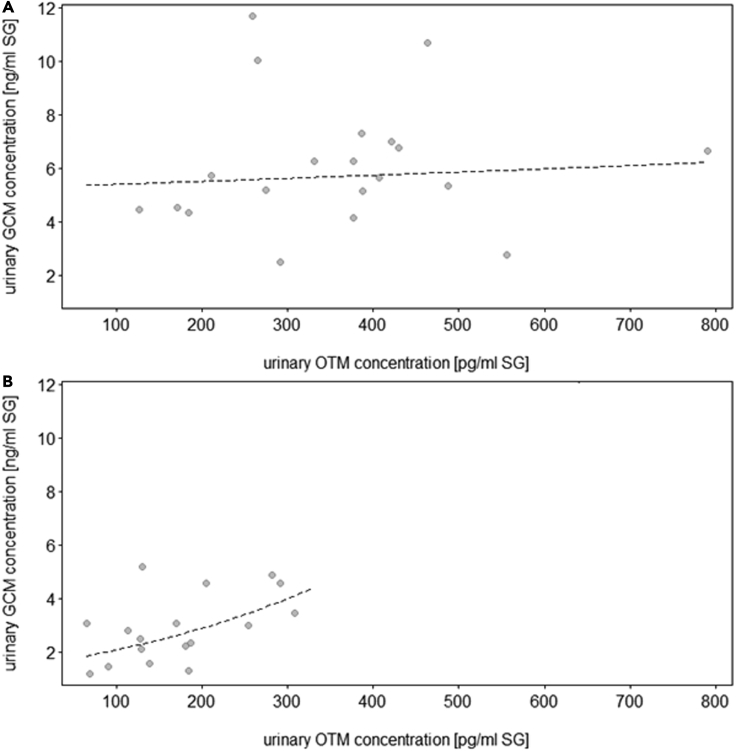
Link between oxytocin and glucocorticoids (control condition) (A and B) Effect of urinary OTM on urinary GCM concentrations (ng/mL, corrected for specific gravity, SG) in a) dogs (N = 10 individuals) and b) wolves (N = 9 individuals). The dashed line represents the fitted model of the effect of urinary OTM on urinary GCM concentrations following the control condition (baseline samples).

**Figure 8:**
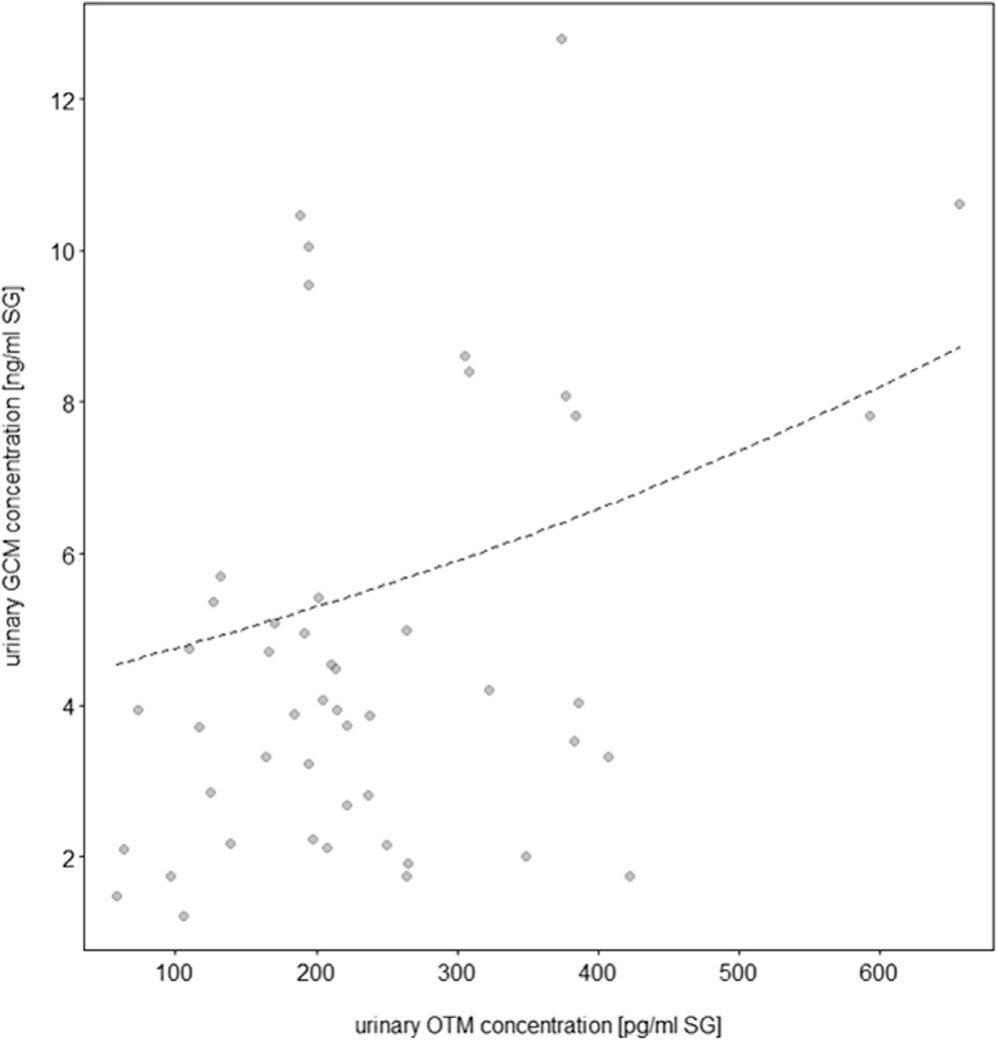
Link between oxytocin and glucocorticoids (chorus howling condition) Effect of urinary OTM on urinary GCM concentrations (ng/mL, corrected for specific gravity, SG) The dotted line represents the fitted model of the effect of urinary OTM on urinary GCM concentrations following the induced chorus howling condition in both dogs and wolves.

In the control condition, uOTM concentrations contributed significantly to uGCM concentrations in wolves but not dogs, i.e., wolves with higher uOTM also had higher uGCM concentrations (LRT = 4.6, df = 1, p = 0.032; Figures 7A and 7B, Table S14, supplemental information). This was also the case in the chorus howling condition, but regardless of species (LRT = 3.7, df = 1, p = 0.054; Figure 8, Table S15, supplemental information). Urinary OTM and uGCM were not linked in the territorial (LRT = 0.7, df = 1, p = 0.393) and separation (LRT = 0.9, df = 1, p = 0.330) conditions, in either species.

## Discussion

The aim of the present study was to compare wolves’ and dogs’ behavioral and hormonal correlates of conspecific interactions in non-feeding contexts. In contrast to our predictions, we found relatively few behavioral differences between wolves and dogs. However, interesting differences between the two species emerged in relation to the association between synchronized movement, territorial behavior, and hormonal correlates, and in the response to separation from their pack mates. In addition, the correlation between uOTM and uGCM concentrations was context- and species-specific.

Both wolves and FRD maintain and patrol their territorial borders, engaging in potentially violent, sometimes lethal, intergroup conflicts to defend them.^12^^,^^27^ We did not find differences between dogs and wolves in the proportions of territorial behaviors exhibited during the territorial condition, but a positive association between urinary OTM and GCM concentrations and territorial behavior emerged only in wolves (Figures 3A and 3B, Figures 5A and 5B). However, urinary OTM and GCM concentrations were not associated with each other following the territorial condition. Independent activity of the oxytocinergic system and HPA axis was previously described in chimpanzees in the context of intergroup conflicts whereby the magnitude of out-group threat affected only cortisol but not OT secretion.^59^ Hence, the activation of the OT system in response to an out-group threat may serve to enhance in-group coordinated and cohesive behavior rather than promote out-group hostility.^76^ This is supported by research in rodents, where OT treatment modulated the physiological and behavioral stress response to a threat by increasing conspecific affiliation.^80^ In the present study, wolves showed a more pronounced hormonal response to an out-group threat than dogs in line with their different socio-ecologies. Wolf inter-pack encounters pose a lethal risk, whereby packs are more likely to chase off rivals if their own pack outnumbers the rivaling pack.^81^ Accordingly, within-pack social bonds need to be strong to ensure that everyone cooperates when confronted with an out-group threat, making the reinforcement of group cohesion essential to survival.^81^^,^^82^ FRDs, on the other hand, do not rely on extensive conspecific cooperation for hunting and breeding, and territorial conflicts appear to be less serious than in wolves.^28^ Hence, the lack of a strong physiological response to territorial threats may be related to a more relaxed need for group cohesion in dogs, or changes brought about by domestication, whereby dogs’ reactivity to outgroup threats may have been selected against, making them more tolerant.

In line with our predictions, there was a link between synchronized locomotion and uOTM concentrations in wolves but not in dogs (Figures 2A and 2B). Although dogs showed higher proportions of synchronized movement during the territorial condition than wolves, it was not related to higher uOTM concentrations. Rather, wolves synchronized their behavior more than dogs during the induced chorus howling condition, supporting the idea that the oxytocinergic system plays a role in strengthening intra-pack coordination in wolves. Surprisingly though, chorus howling, which was more prevalent in wolves than dogs, was not linked to OT release. This may be because of a couple of important limitations: In the present study, chorus howling was elicited by an external acoustic stimulus (‘induced howling’) and thus the emotional valence of the howling response may differ from spontaneous chorus howling.^83^ However, chorus howling also occurred spontaneously multiple times in the wolf packs. Yet, as for induced chorus howling, there was no link between spontaneous howling and urinary OTM concentrations. Nevertheless, there might have been other cues which triggered howling that we remained unaware of. It has been shown that wolf howls’ acoustic parameters vary according to behavioral context.^83^ Hence, a more sophisticated analysis of the acoustic structure of howls would be necessary to recognize subtle differences between them which, in turn, may be linked to differences in underlying motivation to howl, and differences in physiological arousal.

Although not a main variable of interest in the present study, we recorded barking from four different wolves (2 males, 2 females). As for the male wolves, they barked only during the separation condition when left alone in their home enclosures. The duration of barking was very short, between 2 and 6 s (average 3.5 s). One of the females also barked only during separation on two occasions, for an average of 28.5 s. The other female barked during chorus howling, on two occasions, for an average of 3 s. In contrast with the wolves, all the dogs barked during the study, and we recorded dog barking in all test conditions. Dogs barked the most during separation from their pack (on average for 126 s), but also during the territorial condition (on average for 47 s), chorus howling (on average for 35 s), and control condition (4 s). Hence, most of the barking occurred during separation from the pack which was linked to elevated GC concentrations and elicitation of a stress response only in wolves. This is intriguing, because barking in wolves is thought to occur primarily in threatening contexts such as territorial defense or dominance interactions. Because the experimenter who recorded the videos was present in every condition, it is not possible to state whether barking was associated with human presence or not. It appears, though, that further in-depth research into how the domestication process and social ecology have shaped canid vocal communication, particularly barking and howling, is needed.

Social isolation is a strong stimulus of emotional, psychological, and physiological distress.^84^ Accordingly, separation distress resulting from the disruption of a close social bond is thought to promote behaviors that facilitate reunion,^85^ thus representing an essential mechanism for individuals that depend on their group for survival. Previous work on captive wolves and dogs found that relationship quality and rank distance predicted the magnitude of the behavioral and physiological stress response to separation.^17^^,^^65^ Specifically, wolves exhibited more escape attempts and stress-related behaviors when left behind by their pack mates than dogs.^17^ In the present study, although no significant differences in wolves’ compared to dogs’ behavioral responses to separation emerged on group level, several wolves but no dogs, showed high levels of stress-related behaviors such as pacing, biting into the enclosure fence, and attempting to dig their way out of the enclosure. Wolves also moved around more during separation, seemingly searching for their pack mates, whereas dogs behaved in a more passive manner. As predicted, wolves but not dogs had increased GCM concentrations following separation (Figure 4). Furthermore, wolves solo howled longer than dogs during separation, which was positively linked to uGCM concentrations in both species (Figure 6), as were escape-related behaviors (Figure S2; it should be noted that this effect appears to be driven by just one individual wolf that had extraordinarily high uGCM concentrations). Taken together, despite both wolves and dogs showing behavioral indications of separation distress, the underlying emotional and physiological state appeared to be one of greater arousal in wolves than dogs. Given the importance of pack cohesion for wolves whose individual survival depends on cooperation, these results are in line with predictions arising from the SE hypothesis.^6^

Finally, we found a positive association between urinary OTM and GCM concentrations in the control and the induced chorus howling conditions (Figures 7A, 7B, and 8). The first finding was specific to wolves, in line with previous data from this population.^35^ The latter, however, was not species-specific; instead, following chorus howling, both wolves and dogs showed a positive correlation of uOTM and uGCM concentrations. This may suggest that chorus howling is related to physiological arousal in wolves and dogs, reflected in simultaneous activation of the oxytocinergic system and HPA axis. However, given the lack of a correlation between howling and uOTM concentrations as outlined above, this finding must be interpreted with caution and requires further investigation to pinpoint the relationship between group vocalization and hormonal parameters. In line with our predictions regarding the stress buffering effect of social partner presence, there was no correlation between uOTM and uGCM in the separation condition. However, in contrast to the predictions, there was no correlation also in the territorial condition, although both uOTM and uGCM concentrations were affected by territorial behavior in wolves. It appears that the relationship between oxytocinergic and HPA axis activity is context-as well as species-dependent but more in-depth investigation is required if we want to move from describing correlational evidence to defining causal links.

In summary, uOTM concentrations were higher in dogs than wolves, but unaffected by experimental condition. Furthermore, we found that males of both species had higher uOTM concentrations than females. This is partly in line with our previous work where male dogs were found to have higher uOTM levels than all other groups.^35^ As of now it is unclear why this may be the case, but ongoing work in our group is investigating potential links between the oxytocinergic system and canid reproductive behavior and physiology, specifically, gonadal steroid levels.

Dogs had higher uGCM concentrations than wolves in the control condition, in line with previous studies (urine^35^; saliva^69^), but at odds with the hypothesis that domesticated canids should have lower GC concentrations than their wild relatives.^38^^,^^66^ Interestingly, dogs showed fewer changes in uGCM concentrations, i.e., lower HPA axis reactivity, across conditions than wolves (Figure S3, lower panel), which aligns with evidence from comparisons of wild and domesticated animals.^67^^,^^68^ Indeed, during forced separation from their pack, wolves appeared actively trying to follow their pack members, pacing, and moving around in the enclosure, whereas dogs seemed to passively wait for their companions’ return. This was reflected in a higher endocrine stress response in wolves than dogs (differences in locomotor activity were accounted for). It is plausible that, over the course of domestication, the need to affiliate and cooperate with conspecifics grew less important to dogs’ survival. In summary, our data demonstrate that wolves and dogs show only subtle behavioral differences but differ in their physiological responses to group activities and separation. Indeed, considering only behavioral measures, wolves and dogs did not differ in the overall amount of synchronization with their pack mates, nor the proportion of territorial or escape-related behaviors they showed. Yet, endocrine parameters differed. This highlights an important aspect for future comparative studies: Behavioral data alone may not adequately reflect differences in physiological and emotional processes. Hence, whenever possible, behavioral studies should be complemented by physiological measures.

To conclude, the present study provides evidence for limited behavioral differences between hand-raised, group housed wolves and dogs but an alteration in the association of behavioral and endocrine responses to conspecific interactions. Specifically, in wolves but not in dogs, synchronized movement was positively related to OT concentrations, territorial behavior was positively related to OT and GC release, and separation from the pack was related to increased GC concentrations. These findings are in line with species-specific socioecological constraints that require higher levels of intra-pack coordination, resulting in greater cohesiveness and dependence on their pack members, in wolves compared to dogs. Given wolves and dogs in this study were raised and kept under similar conditions, differences between them can be attributed to changes brought about by the domestication process rather than ontogeny. In the future, similar studies should be performed on different populations of free-ranging animals (both dogs and wolves) to validate their significance under natural conditions.

### Limitations of the study

Finally, an important limitation of the present study concerns the number of animals per group available at our study site which varied between 2 and 4 animals. Although we included the random intercept effect of pack id into our statistical models which accounted for random variability between different packs and dyads, we were unable to estimate the fixed effect of group size on response variables without further increasing model complexity. Indeed, the number of social partners in the group as well as indices of group composition such as sex, age, and ranks of the members, may affect social interactions in canids, specifically related to territoriality.^12^ Ultimately, the present study provides a first comparison of wolf/dog behavioral endocrinology in a relatively controlled, comparative setting and small, “artificial” packs (i.e., all packs remained stable during the study period but were initially formed by co-housing and/or introducing hand-raised pups to already existing packs). Whether results hold in larger groups and natural populations remains to be investigated.

## STAR★Methods

### Key resources table

**Table:** 

REAGENT or RESOURCE	SOURCE	IDENTIFIER
**Antibodies**		

Antibody against cortisol-21-hemisuccinate linked to BSA	Unit of Physiology, Pathophysiology and Experimental Endocrinology, Department of Biomedical Sciences, University of Veterinary Medicine, Veterinaerplatz 1, 1210 Vienna, Austria	Antibody B described in Zeugswetter et al.^94^

**Biological samples**		

Urine samples of wolves (*Canis lupus*) and dogs (*Canis lupus familiaris*)	Wolf Science Center Austria	https://www.wolfscience.at

**Critical commercial assays**		

Arbor Assays Oxytocin ELISA kit	Arbor Assays, Ann Arbor, Michigan, USA	CAT #K048-H5 https://www.arborassays.com/product/oxytocin-enzyme-immunoassay-kit/

**Deposited data**		

Dataset	This study	Wirobski, Gwendolyn (2023), “Dataset for statistical analyses”, Mendeley Data, V2, https://doi.org/10.17632/ky2vybch4z.2
R code	This study	Wirobski, Gwendolyn (2023), “R code”, Mendeley Data, V2, https://doi.org/10.17632/6v496pf3r2.2

### Resource availability

#### Lead contact

Further information and requests for resources and reagents should be directed to and will be fulfilled by the lead contact, Gwendolyn Wirobski (gwendolyn.wirobski@vetmeduni.ac.at).

#### Materials availability

This study did not generate new unique reagents.

### Experimental model and subject details

#### Animals

Ten adult mongrel dogs (5 males, 5 females; 4–9 years old) and 10 adult grey wolves (5 males, 5 females; 3–10 years old) housed in conspecific dyads (dogs: two dyads; wolves: four dyads) or packs of up to three (dogs: one pack of three; wolves: two packs of three) or four (dogs: one pack of four) animals at the Wolf Science Center (WSC) Austria (www.wolfscience.at/en), participated in this study. Packs remained stable throughout the study period. As individuals from different packs do not behave in a friendly manner towards each other, they are always kept in separate enclosures and do not have direct contact. Not all animals living at the WSC could be tested in this study because we were unable to habituate some of the individuals to the urine sample collection procedure. One male wolf provided only a subset of samples due to a recurring bladder infection during the testing period. All animals were hand-raised by animal professionals from an early age and introduced into conspecific packs at the age of approx. 5 months. Dogs were provisioned daily with commercial dry dog kibble and received enrichment in form of meat and pieces of sausage at least once a week and during training sessions. Wolves were fed every 2–3 days with raw meat and carcasses (mainly deer, chicken, and rabbit). All animals had *ad libitum* access to drinking water. Male individuals were vasectomized at approx. 6 months of age to prevent reproduction but maintain their hormonal and behavioural profiles. We avoided testing during the animals’ breeding season.

#### Ethics statement

This study was approved by the institutional ethics and animal welfare committee in accordance with Good Scientific Practice (GSP) and ARRIVE guidelines and national legislation (approval number ETK 08/05/2018, University of Veterinary Medicine Vienna, Austria).

### Method details

#### Experimental conditions

We employed a within-subject design whereby each animal acted as its own control. Testing was conducted pseudo-randomized and counterbalanced to prevent order effects. We did not test wolves during the wolf breeding season (December to March). Further, we did not test dog packs if females showed physical or behavioural signs of oestrus. To minimise disturbances or distractions before and during testing, all staff members were instructed not to feed, interact with, or clean enclosures of focal packs for at least 2 hours before testing began. Each test condition was repeated up to three times per animal to allow us to include within-individual differences (i.e., daily fluctuations in behaviour and hormone concentrations) into the statistical models. There were three test and one control condition (Figures 1A–1H).

#### Mock territorial patrol

To initiate territorial behaviour experimentally, we created a mock territorial patrol situation: At the beginning of the test, an animal trainer shifted the focal pack from their home into an adjacent test enclosure which had before been occupied by a different pack. Specifically, the other pack had stayed in the test enclosure on the same day for at least two hours before the test in full view of the focal pack. The focal pack was then left to explore the enclosure for 60 min while the other pack remained in proximity (separated by two rows of fencing). Hence, the animals were able to smell, hear and see each other for the duration of the test. The focal pack’s behaviour was recorded on video, and the animals were taken out for urine collection walks 60 min after the start of the test (for detailed description and rationale of the timeline of sample collection, see below).

#### Induced chorus howling

This condition took place on Saturdays at noon (12 am) when a fire siren regularly went off nearby (i.e., weekly test alarm). This event reliably elicited chorus howling in the wolves and dogs at the WSC. We started the focal packs’ observation 15 min before the onset of the siren and recorded the animals’ behaviour for 60 min after the howling event. If the focal pack did not howl, it was observed again the next week. As in the other conditions, the focal animals were taken out on leashed walks to collect urine samples 60 min after howling onset. In addition, we also observed 19 occurrences of spontaneous (i.e., no apparent external trigger identifiable) chorus howling in the wolves (but only once in the dogs) and opportunistically collected urine samples after 60 min.

#### Separation from the pack

For the separation condition, the focal animal remained in the home enclosure while its pack members were taken on walks or to a different enclosure out of sight where they remained for the whole test duration (60 min). The focal individual’s behaviour was recorded on video, and it was taken out for urine collection 60 min after the start of the separation.

#### Unsolicited period in the pack (control condition)

We repeatedly observed and sampled all individuals following 60 min of an undisturbed period with their pack in their home enclosures to obtain unstimulated (basal) samples for comparison of hormone concentrations. This approach was chosen over pre-post sampling to avoid taking out animals on urine collection walks repeatedly and thereby potentially affecting their subsequent behaviour and hormonal responses to the experimental conditions. In addition, we compared the animals’ behaviour during the experimental conditions to the control condition to determine whether our experimental design successfully elicited behavioural changes.

#### Behavioural data collection

Focal animals were filmed, and durations and frequencies of behaviours (full ethogram, Table 1) were subsequently coded using Solomon video coding software (version beta 17.03.22, copyright András Péter). All behaviours recorded as durations were normalized for the observation time the animal was in sight (i.e., visible on video) and expressed as proportions of total time in sight for statistical analyses. Inter-observer-reliability (IOR) scoring was conducted by two independent coders and calculated using the package ‘irr’ (version 0.84.1) in R, version 4.1.1.^86^ This revealed overall excellent to good reliability: locomotion, interclass correlation coefficient (ICC) = 0.98, p< 0.01, 95% confidence intervals (CI) 0.73–0.99; affiliative behaviours, ICC = 0.99, p< 0.01, CI 0.98–0.99; synchronized behaviours, ICC = 0.93, p< 0.01, 95% CI 0.69–0.98; territorial behaviour, ICC = 0.95, p< 0.01, 95% CI 0.78–0.99; chorus howling, ICC = 0.72, p< 0.05, 95% CI 0.15–0.94; solo howling, ICC = 0.74, p< 0.05, 95% CI 0.10–0.94; escape/distress-related and agonistic behaviours did not occur often enough for ICC calculation.

#### Urine collection, extraction, and hormone measurement

OT released into the periphery from the posterior pituitary gland is transported through the bloodstream and cleared by the kidneys into the urinary bladder.^87^^,^^88^^,^^89^ In dogs, urinary OT concentrations reach peak levels within 45–60 minutes after a trigger event^90^ and elevated urinary GCM concentrations have been measured between 60–90 minutes following a stressor in dogs and wolves.^35^^,^^91^ Therefore, we took animals out on urine collection walks 60 min after the start of the experimental condition. Urine samples were obtained non-invasively using an expandable metal stick with a plastic sampling cup attached to it.^35^ All participating animals were previously habituated to this procedure and showed no signs of distress during sample collection. Within 15 min of collection, urine samples were split into 1 mL aliquots and 100 μl of a 0.5 N phosphoric acid (PA) was added to lower the pH and prevent OT degradation.^92^ No PA was added to samples used for uGCM measurement. All samples were frozen at −20°C until extraction and measurement.

Solid-phase extractions (SPE) were performed for uOTM measurement according to a previously validated protocol for wolf/dog urine samples.^92^ In brief, the samples were thawed, vortexed, and centrifuged (1 min, 365*g*, 4°C). SPE cartridges (Chromabond HR-X, 30 mg, 1 mL, Macherey-Nagel, Dueren, Germany) were conditioned with 1 mL methanol (100%, HPLC grade) followed by 1 mL HPLC water on a vacuum chamber (Chromabox, Macherey-Nagel). Cartridges were loaded with 0.5 mL of the sample and diluted with 0.5 mL buffer solution (water, 0.1% triflouroacetic acid (TFA)). The subsequent washing step consisted of adding 5 mL wash buffer (10% (vol/vol) acetonitrile (ACN) containing 1% TFA in water) to each cartridge. Then, the cartridges were dried using a vacuum pump and samples were eluted with 1 mL 80% (vol/vol) ACN into fresh glass tubes. Finally, eluted samples were evaporated until completely dry, at 50 °C for 35 min using a stream of air and reconstituted in 0.3 mL 100% ethanol. For uOTM measurement, samples were shipped to the Max Planck Institute for Evolutionary Anthropology (MPI EVA), Leipzig, Germany. Shipment took less than 10 hours and samples were kept on dry ice throughout. All samples were measured in duplicates using a commercially available oxytocin assay kit (Arbor Assays, Ann Arbor, Cat. No: K048-H5). Prior to the study, the kit was analytically and physiologically validated for both species and substrate.^93^ Sample measurement was repeated when optical density (OD) values of duplicates differed more than 10% or when the measurement fell below or above the linear range of the assay. The standard curve ranged from 16.4 to 10,000 pg/mL and assay sensitivity was 17.0 pg/mL. The cross-reactivities at 50% binding point, as reported in the kit manual, were 94.3% for isotocin, 88.4% for mesotocin, 0.14% for lys-vasopressin, 0.13% for arg-vasotocin, and 0.12% for arg-vasopressin. The intra-assay coefficient of variation (CV) was 10% (averaged across duplicates of all samples on one plate). Inter-assay CVs were 7% for a high concentrated OT standard (640 pg/mL) and 10% for a low concentrated OT standard (102.4 pg/mL).

For uGCM measurement, samples were extracted with diethyl-ether: Briefly, samples were thawed, vortexed, and 0.5 mL was pipetted into a clean 10 mL glass tube before 5 mL diethyl-ether was added. The tubes were then vortexed again, centrifuged (15 min, 2500*g*), capped and stored at −20°C for at least 5 hours. Then, the supernatant organic phase was transferred to a new glass tube and evaporated until dry. To each tube, 0.5 mL of assay buffer was added. Tubes were left at room temperature for 10 minutes and then vortexed before sealed and stored at −20°C until uGCM measurement using an in-house cortisol assay (antibody B against cortisol-21-hemisuccinate linked to BSA^94^), biologically validated for our purpose^35^ at the Unit of Physiology, Pathophysiology, and Experimental Endocrinology, University of Veterinary Medicine, Vienna, Austria. The assay standard curve ranged from 2 to 200 pg/well. Assay sensitivity was 2 pg/well. Intra- and inter-assay CVs based on samples were 5% and 8%, respectively.

To control for variable water content in the samples, we measured the specific gravity (SG) of each sample with a digital refractometer (TEC++, serial no. T6017). Hormone concentrations were calculated using the formula given in Miller et al.^95^ and expressed as pg/mL SG (uOTM) and ng/mL SG (uGCM).

### Quantification and statistical analysis

The total sample size used for statistical analyses was N = 180 (78 dog samples, 102 wolf samples). To test whether wolves and dogs showed behavioural differences during the test conditions, and whether the test conditions elicited differential behavioural reactions compared to the control condition, we fitted generalized linear mixed models (GLMM) with beta error structure in R (version 4.1.1^86^) using the function glmmTMB of the package glmmTMB (version 1.1.2.3^96^). This error distribution was chosen because the dependent variables (response variables) were expressed as proportions (i.e., durations normalized for total time in sight). The interaction between species and condition was included in each model as the test predictor, and sex as a control predictor. Further, random intercept effects of subject and pack id were included.

To investigate whether hormone concentrations differed between test conditions in wolves and dogs, we fitted linear mixed models (LMM) with Gaussian error structure in R (version 4.1.1^86^) using the function lmer of the R package lme4 (version 1.1-27.1^97^) with the optimizer ‘bobyqa’. The response variables (uOTM and uGCM concentrations) were log-transformed to obtain normally distributed and homogenous residuals. The interaction between species and condition was included as the test predictor. Feeding status (as a factor with two levels: fed, fasted; only applicable for wolves as dogs were fed daily), reproductive phase (as a factor with two levels: anestrus, diestrus, as no tests were performed during proestrus and oestrus phases), normalized duration of locomotion (co-variate, z-transformed to facilitate interpretation), and sex interacting with species were included as control predictors since they can affect uGCM and uOTM concentrations in wolves and dogs.^35^Further, we included random effects of subject, pack, and assay plate id to account for repeated, non-independent samples and variation between subjects, packs, and plates.

Then, we explored whether hormone concentrations correlated with observed behaviours, and whether this differed between dogs and wolves. To this end, we fitted LMMs with Gaussian error structures and uOTM/uGCM concentrations as the response variables. Normalized durations (i.e., as proportions of total observation time in sight) of synchronized movement, escape/distress-related, territorial behaviour, as well as solo howling, whining, and chorus howling interacting with species were included as test predictors in the respective models. Further, all control predictors mentioned above (sex, feeding status, and reproductive phase) and the normalized duration of locomotion were included as control variables. Subject, pack, and assay plate id were added as random effects.

Since we were able to record 19 incidences of spontaneous chorus howling in wolves and collect the corresponding urine samples, we fitted a separate model to test whether spontaneous howling would be associated with uOTM concentrations in wolves. This model contained the normalized duration of chorus howling as the test predictor and all control factors mentioned above, including all random effects.

Finally, the correlation between uOTM and uGCM concentrations in wolves and dogs in each condition was tested with LMMs, including the interaction between species and uOTM concentrations as the test predictor, and subject, pack, and plate id as random intercept effects.

All full models were tested against a null model lacking the test predictor (i.e., the interaction and its main effects) but retaining all control and random effects^98^ using a likelihood ratio test.^99^ If the full-null model comparison was not significant, no further tests were performed. In case an interaction term did not reveal significance, we fitted a reduced model lacking the interaction but containing the main effects. Diagnostic plots (residuals vs fitted and qqplot) to examine assumptions of normality and homogeneity of variances for each model. We assessed model stability by comparing estimates obtained from the model based on all data with those obtained from models with the levels of the random effects excluded one at a time. Collinearity was assessed using the function ‘vif’ of the package car (version 3.0-11) and revealed no higher values than 2.4, indicating collinearity was not an issue. To obtain confidence intervals we performed parametric bootstrapping (function bootMer of lme4). Where applicable (i.e., significant interaction term), post hoc pairwise comparisons were performed using the package lsmeans (version 2.30-0^100^). Output tables for all models that revealed significance in the full-null model comparison including model stability estimates and confidence intervals for each predictor can be found in the supplemental information.

## Data Availability

• Data used to fit models reported in this paper have been deposited at Mendeley and are publicly available as of the date of publication. DOI listed in the key resources table. • All original code has been deposited at Mendeley and is publicly available as of the date of publication. DOI listed in the key resources table. • Any additional information required to reanalyse the data reported in this paper is available from the lead contact upon request.
