# Supplementary material for: Similar behavioral but different endocrine responses to conspecific interactions in hand-raised wolves and dogs
Source: iScience. 2023 Jan 14;26(2):105978. doi: 10.1016/j.isci.2023.105978 (PMC9900400; doi:10.1016/j.isci.2023.105978)
Supplement: Document S1. Figures S1–S3 and Tables S1–S15 [file mmc1.pdf]

iScience, Volume *26*

Supplemental information

Similar behavioral but different endocrine responses to conspecific interactions in hand-raised wolves and dogs

Gwendolyn Wirobski, Friederike Range, Evelien A.M. Graat, Rupert Palme, Tobias Deschner, and Sarah Marshall-Pescini

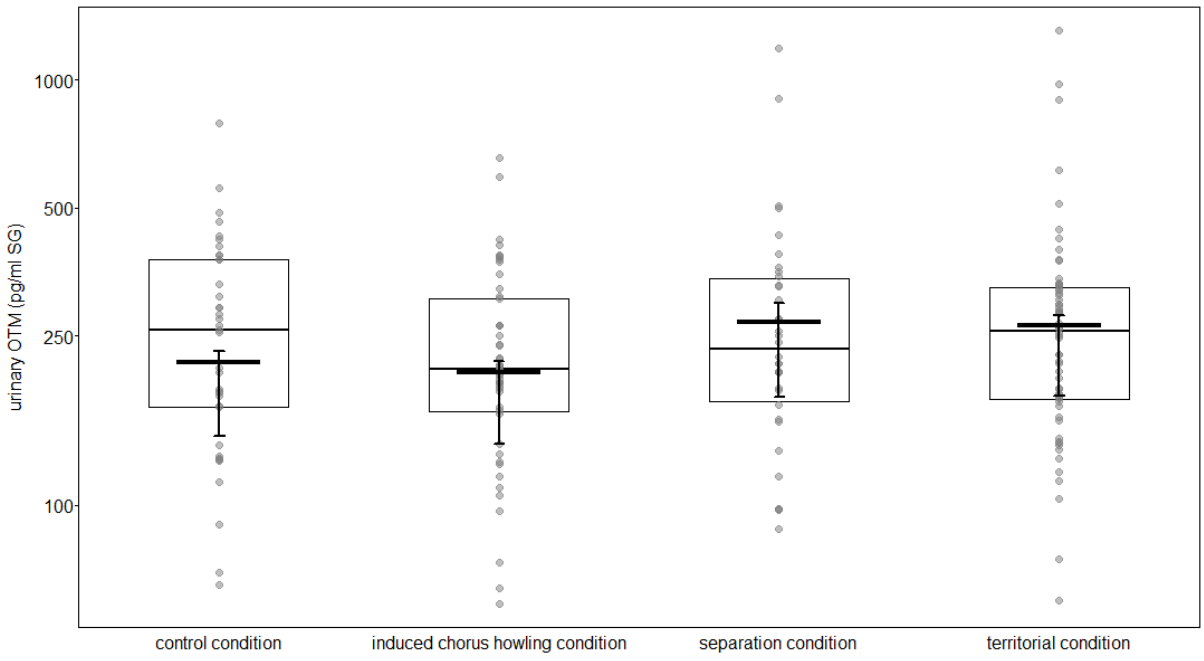

Fig. S1. Urinary OTM concentrations (pg/ml SG) of wolves and dogs across conditions. Indicated are medians and quartiles (horizontal lines with boxes) as well as the fitted model and its 95% confidence intervals (thick horizontal lines with error bars). Grey dots represent individual samples. Related to Fig. 4.

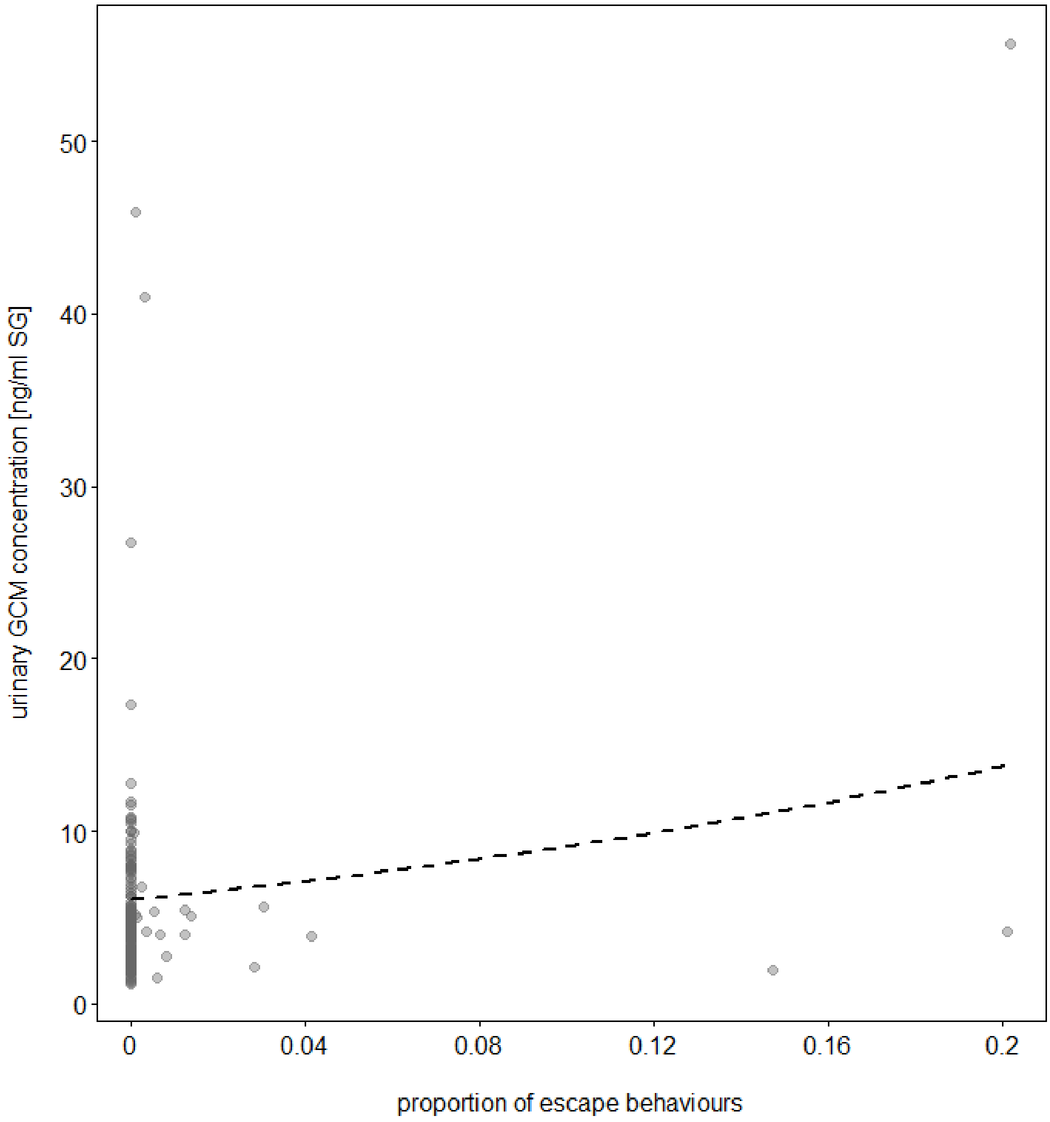

Fig. S2: Association of escape-related behaviours and urinary GCM concentrations (ng/ml, corrected for specific gravity, SG) in dogs (N=10 individuals) and wolves (N=9 individuals). The dashed line represents the fitted model for the effect of escape behaviours on urinary GCM concentrations, given all control predictors and random effects. Each dot represents a sample. Every individual provided 1-3 samples. The model accounted for repeated sampling. Related to Fig. 4.

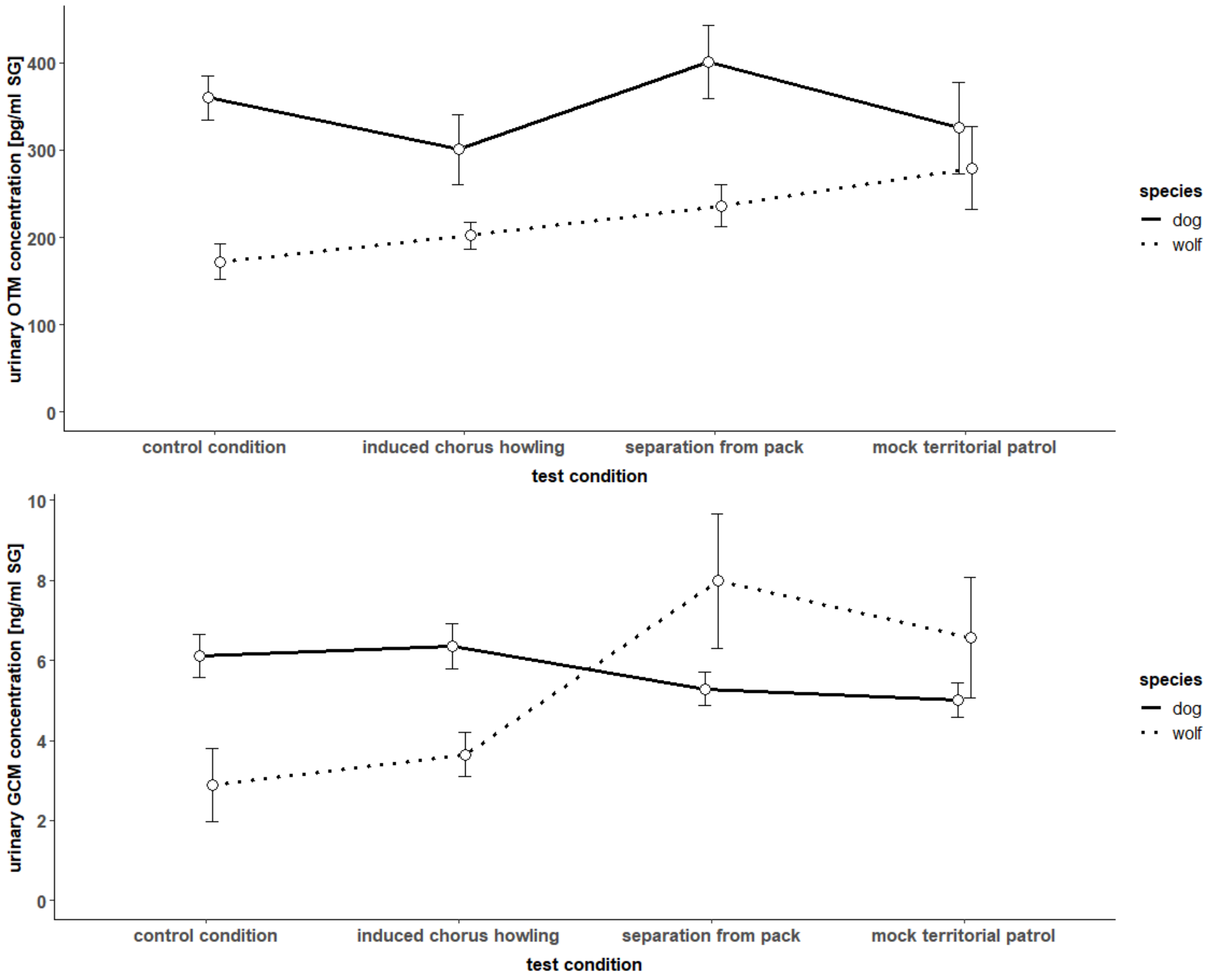

Fig. S3. Comparison of urinary OTM and GCM concentrations across test conditions in dogs (solid line) and wolves (dotted line). The graph shows means and standard error bars. Related to Fig. 1 A-H.

Table S1: Full and reduced model outputs for the effects of species and condition on urinary OTM (pg/ml SG; log transformed) concentrations. Related to Fig. S1 and STAR Methods.

**Full model** / **Predictor**	**Estimate**	**SE**	**Df**	**χ 2**	**P**
Intercept	5.935	0.184	NA*	NA*	NA*
Species†	-0.632	0.188	NA*	NA*	NA*
Induced chorus howling condition‡	-0.173	0.118	NA*	NA*	NA*
Separation condition‡	0.170	0.125	NA*	NA*	NA*
Territorial condition‡	0.019	0.143	NA*	NA*	NA*
Sex§	0.210	0.097	NA*	NA*	NA*
Feeding status¶	-0.589	0.082	1	13.946	**0.000**
Reproductive phase#	-0.001	0.116	1	0.000	0.996
Locomotion°	-0.040	0.042	1	0.742	0.389
Species†: Induced chorus howling condition‡	0.227	0.163	3	4.006	0.261
Species†: Separation condition‡	0.043	0.166	NA*	NA*	NA*
Species†: Territorial condition‡	0.290	0.171	NA*	NA*	NA*
Species†: Sex§	-0.026	0.131	1	0.029	0.865

**Reduced model** / **Predictor**	**Estimate**	**SE**	**Df**	**χ 2**	**P**	**Lower CI**	**Upper CI**	**Stability min**	**Stability max**
(Intercept)	5.856	0.173	NA*	NA*	NA*	5.513	6.212	5.719	5.967
Species†	-0.496	0.136	1	8.670	**0.003**	-0.784	-0.225	-0.574	-0.414
Induced chorus howling condition‡	-0.053	0.082	3	7.205	0.066	-0.211	0.096	-0.108	0.021
Separation condition‡	0.187	0.089	NA*	NA*	NA*	0.007	0.356	0.116	0.238
Territorial condition‡	0.184	0.112	NA*	NA*	NA*	-0.032	0.403	0.061	0.283
Sex§	0.192	0.066	1	4.878	**0.027**	0.051	0.319	0.135	0.230
Locomotion	-0.059	0.041	1	1.731	0.188	-0.141	0.022	-0.104	0.000
Feeding status¶	-0.589	0.085	1	14.135	**0.000**	-0.756	-0.427	-0.656	-0.502
Reproductive phase#	-0.004	0.114	1	0.001	0.971	-0.232	0.261	-0.167	0.085

Statistically significant results ($P \leq 0.05$) appear in bold. SE, standard error. Df, degrees of freedom. CI, confidence interval. * Not shown because of limited interpretation only.

†, ‡, §, ¶, # χ^2 and P values refer to comparison with the test predictors' reference levels:

† Reference level 'dog'.

‡ Reference level 'control condition'.

§ Reference level 'female'.

¶ Reference level 'not fed/fasted'.

# Reference level 'anestrus'.

° Co-variate (normalized duration, z-transformed).

Table S2: Full model output for the effect of species and condition on the proportions of synchronized movement. Related to STAR Methods.

Full model<br>Predictor	Estimate	SE	Df	χ 2	P	Lower CI	Upper CI	Stability min	Stability max
Intercept	-4.038	0.282	NA*	NA*	NA*	-4.038	-4.639	-4.358	-3.911
Species†	-0.063	0.371	NA*	NA*	NA*	-0.063	-0.786	-0.186	0.277
Induced chorus howling‡	0.306	0.244	NA*	NA*	NA*	0.306	-0.159	0.087	0.450
Territorial condition‡	2.636	0.195	NA*	NA*	NA*	2.636	2.255	2.566	2.686
Sex§	-0.220	0.142	1	2.076	0.150	-0.220	-0.512	-0.320	-0.113
Species†: Induced chorus howling‡	0.040	0.340	2	11.919	**0.003**	0.040	-0.687	-0.120	0.258
Species†: Territorial condition‡	-0.722	0.295	NA*	NA*	NA*	-0.722	-1.308	-0.877	-0.598

Statistically significant results ($P \leq 0.05$) appear in bold. SE, standard error. Df, degrees of freedom.
CI, confidence interval. * Not shown because of limited interpretation only.
†, ‡, § χ^2 and P values refer to comparison with the test predictors' reference levels:
† Reference level 'dog'.
‡ Reference level 'control condition'.
§ Reference level 'female'.

Table S3: Full model output for the effect of species and synchronized movement on urinary OTM (pg/ml SG; log transformed) concentrations. Related to Fig. 2 and STAR Methods.

Full model<br>Predictor	Estimate	SE	Df	χ 2	P	Lower CI	Upper CI	Stability min	Stability max
Intercept	5.954	0.155	NA*	NA*	NA*	5.628	6.254	5.857	6.062
Species†	-0.488	0.137	NA*	NA*	NA*	-0.752	-0.200	-0.609	-0.388
Synchronized movement°	-0.038	0.045	NA*	NA*	NA*	-0.119	0.054	-0.088	-0.013
Sex§	0.205	0.063	1	5.949	**0.015**	0.069	0.343	0.144	0.234
Feeding status¶	-0.575	0.077	1	13.985	**0.000**	-0.720	-0.418	-0.628	-0.488
Reproductive phase#	0.025	0.112	1	0.046	0.830	-0.205	0.244	-0.145	0.160
Locomotion°	0.010	0.039	1	0.067	0.796	-0.064	0.089	-0.021	0.071
Species†: Synchronized movement°	0.147	0.073	1	3.880	**0.049**	0.001	0.287	0.094	0.243

Statistically significant results ($P \leq 0.05$) appear in bold. SE, standard error. Df, degrees of freedom.
CI, confidence interval. * Not shown because of limited interpretation only.
†, ‡, §, ¶, # χ^2 and P values refer to comparison with the test predictors' reference levels:
† Reference level 'dog'.
§ Reference level 'female'.
¶ Reference level 'not fed/fasted'.
# Reference level 'anestrus'.
° Co-variate (normalized duration, z-transformed).

Table S4: Full model output for the effect of species and condition on the proportions of induced chorus howling. Related to STAR Methods.

**Full model**<br>**Predictor**	**Estimate**	**SE**	**Df**	**χ 2**	**P**	**Lower CI**	**Upper CI**	**Stability min**	**Stability max**
Intercept	-4.750	0.162	NA*	NA*	NA*	-5.107	-4.462	-4.815	-4.670
Species†	-0.010	0.221	NA*	NA*	NA*	-0.443	0.485	-0.037	-0.002
Induced chorus howling‡	0.661	0.194	NA*	NA*	NA*	0.311	1.054	0.613	0.704
Sex§	-0.113	0.108	1	1.099	0.294	0.340	0.103	-0.145	-0.026
Species†: Induced chorus howling‡	0.755	0.262	1	8.127	**0.004**	0.231	1.256	0.703	0.815

Statistically significant results ($P \leq 0.05$) appear in bold. SE, standard error. Df, degrees of freedom. CI, confidence interval. * Not shown because of limited interpretation only.
†, ‡, § χ^2 and P values refer to comparison with the test predictors' reference levels:
† Reference level 'dog'.
‡ Reference level 'control condition'.
§ Reference level 'female'.

Table S5: Full and reduced model outputs for the effect of species and condition on the proportions of territorial behaviour. Related to STAR Methods.

**Full model**<br>**Predictor**	**Estimate**	**SE**	**Df**	**χ 2**	**P**
Intercept	-4.696	0.288	NA*	NA*	NA*
Species†	0.040	0.327	NA*	NA*	NA*
Territorial condition‡	1.307	0.199	NA*	NA*	NA*
Sex§	-0.045	0.231	1	0.037	0.847
Species†: Territorial condition‡	-0.259	0.273	1	0.894	0.344

**Reduced model**<br>**Predictor**	**Estimate**	**SE**	**Df**	**χ 2**	**P**	**Lower CI**	**Upper CI**	**Stability min**	**Stability max**
(Intercept)	-4.600	0.268	NA*	NA*	NA*	-5.126	-4.124	-4.743	-4.256
Species†	-0.148	0.263	1	0.328	0.567	-0.663	0.375	-0.370	0.064
Territorial condition‡	1.189	0.155	1	35.844	**0.000**	0.939	1.479	1.033	1.382
Sex§	-0.052	0.228	1	0.050	0.822	-0.502	0.377	-0.308	0.098

Statistically significant results ($P \leq 0.05$) appear in bold. SE, standard error. Df, degrees of freedom. CI, confidence interval. * Not shown because of limited interpretation only.
†, ‡, § χ^2 and P values refer to comparison with the test predictors' reference levels:
† Reference level 'dog'.
‡ Reference level 'control condition'.
§ Reference level 'female'.

Table S6: Full model output for the effect of species and territorial behaviour on urinary OTM (pg/ml SG; log transformed) concentrations. Related to Fig. 3 and STAR Methods.

**Full model**<br>**Predictor**	**Estimate**	**SE**	**Df**	**χ 2**	**P**	**Lower CI**	**Upper CI**	**Stability min**	**Stability max**
Intercept	5.942	0.158	NA*	NA*	NA*	5.623	6.253	5.860	6.024
Species[†]	-0.506	0.142	NA*	NA*	NA*	-0.790	-0.195	-0.590	-0.421
Territorial behaviour°	-0.079	0.038	NA*	NA*	NA*	-0.156	-0.001	-0.139	-0.017
Sex[§]	0.216	0.063	1	6.493	**0.011**	0.093	0.337	0.152	0.254
Feeding status[¶]	-0.577	0.076	1	14.048	**0.000**	-0.726	-0.420	-0.630	-0.500
Reproductive phase[#]	0.036	0.103	1	0.113	0.737	-0.190	0.252	-0.134	0.109
Locomotion°	0.032	0.034	1	0.893	0.345	-0.032	0.094	-0.003	0.085
Species[†]: Territorial behaviour°	0.137	0.058	1	5.299	**0.021**	0.031	0.254	0.074	0.220

Statistically significant results ($P \leq 0.05$) appear in bold. SE, standard error. Df, degrees of freedom.
CI, confidence interval. [*] Not shown because of limited interpretation only.
[†, ‡, §, ¶, #] χ^2 and P values refer to comparison with the test predictors' reference levels:
[†] Reference level 'dog'.
[§] Reference level 'female'.
[¶] Reference level 'not fed/fasted'.
[#] Reference level 'anestrus'.
° Co-variate (normalized duration, z-transformed).

Table S7: Full model output for the effects of species and condition on urinary GCM (ng/ml SG; log transformed) concentrations. Related to Fig. 4 and STAR Methods.

**Full model** / **Predictor**	**Estimate**	**SE**	**Df**	**χ 2**	**P**	**Lower CI**	**Upper CI**	**Stability min**	**Stability max**
Intercept	1.755	0.225	NA*	NA*	NA*	1.297	2.207	1.506	1.922
Species†	-0.625	0.264	NA*	NA*	NA*	-1.128	-0.075	-0.872	-0.409
Induced chorus howling condition‡	0.060	0.153	NA*	NA*	NA*	-0.256	0.346	-0.021	0.142
Separation condition‡	-0.105	0.156	NA*	NA*	NA*	-0.405	0.190	-0.243	0.013
Territorial condition‡	-0.187	0.187	NA*	NA*	NA*	-0.560	0.186	-0.380	-0.009
Sex§	0.001	0.216	NA*	NA*	NA*	-0.448	0.449	-0.144	0.188
Feeding status¶	-0.129	0.103	1	1.356	0.244	-0.329	0.074	-0.240	-0.058
Reproductive phase#	0.203	0.114	1	2.932	0.087	-0.037	0.440	0.105	0.300
Locomotion°	-0.005	0.053	1	0.030	0.862	-0.119	0.104	-0.034	0.030
Species†: Induced chorus howling condition‡	0.107	0.208	3	16.555	**0.001**	-0.299	0.535	0.027	0.186
Species†: Separation condition‡	0.772	0.213	NA*	NA*	NA*	0.367	1.191	0.668	0.877
Species†: Territorial condition‡	0.672	0.228	NA*	NA*	NA*	0.207	1.139	0.552	0.874
Species†: Sex§	-0.303	0.292	1	0.958	0.328	-0.939	0.254	-0.478	-0.023

Statistically significant results ($P \leq 0.05$) appear in bold. SE, standard error. Df, degrees of freedom. CI, confidence interval. * Not shown because of limited interpretation only.

†, ‡, §, ¶, # χ^2 and P values refer to comparison with the test predictors' reference levels:

† Reference level 'dog'.

‡ Reference level 'control condition'.

§ Reference level 'female'.

¶ Reference level 'not fed/fasted'.

# Reference level 'anestrus'.

° Co-variate (normalized duration, z-transformed).

Table S8. Results of post hoc pairwise comparisons of the interaction effect between species and condition on urinary GCM concentrations: contrasts by species (p-values adjusted Tukey method for comparing a family of 4 estimates). Related to Fig. 4 and STAR Methods.

**Control condition**	Dogs	Wolves	**Territorial**	Dogs	Wolves
Dogs		P = 0.009	Dogs		n.s.
Wolves			Wolves		
**Induced chorus howling**	Dogs	Wolves	**Separation**	Dogs	Wolves
Dogs		P = 0.025	Dogs		n.s.
Wolves			Wolves		

Table S9. Results of post hoc pairwise comparisons of the interaction effect between species and condition on urinary GCM concentrations: contrasts by condition (p-values adjusted Tukey method for comparing a family of 4 estimates). Related to Fig. 4 and STAR Methods.

**Dogs**	Control condition	Induced chorus howling	Territorial	Separation
Control condition		n.s.	n.s.	n.s.
Induced chorus howling			n.s.	n.s.
Territorial				n.s.
Separation				
**Wolves**	Control condition	Induced chorus howling	Territorial	Separation
Control condition		n.s.	n.s.	P = 0.004
Induced chorus howling			n.s.	P = 0.045
Territorial				n.s.
Separation				

Table S10: Full and reduced model outputs for the effects of species, territorial, and escape/stress-related behaviours on urinary GCM (ng/ml SG; log transformed) concentrations. Related to Fig. 5 and STAR Methods.

**Full model** / **Predictor**	**Estimate**	**SE**	**Df**	**χ 2**	**P**
Intercept	1.801	0.228	NA*	NA*	NA*
Species†	-0.326	0.221	NA*	NA*	NA*
Escape/distress behaviour°	-0.438	0.712	NA*	NA*	NA*
Territorial behaviour°	-0.073	0.054	NA*	NA*	NA*
Solo howling°	0.274	0.373	NA*	NA*	NA*
Whining°	0.009	0.041	NA*	NA*	NA*
Sex§	-0.182	0.148	1	1.365	0.243
Feeding status¶	-0.159	0.106	1	2.073	0.150
Reproductive phase#	0.263	0.125	1	3.904	0.048
Locomotion°	-0.002	0.047	1	-0.001	1.000
Species†: Escape/distress behaviour°	0.537	0.713	1	0.536	0.464
Species†: Territorial behaviour°	0.178	0.081	1	4.785	**0.029**
Species†: Solo howling°	-0.155	0.375	1	0.167	0.683
Species†: Whining°	0.270	0.171	1	2.427	0.119

**Reduced model** / **Predictor**	**Estimate**	**SE**	**Df**	**χ 2**	**P**	**Lower CI**	**Upper CI**	**Stability min**	**Stability max**
(Intercept)	1.816	0.184	NA*	NA*	NA*	1.456	2.195	1.672	1.903
Species†	-0.398	0.176	NA*	NA*	NA*	-0.762	-0.026	-0.622	-0.276
Territorial behaviour°	-0.078	0.055	NA*	NA*	NA*	-0.191	0.036	-0.173	-0.038
Escape/distress behaviour°	0.100	0.040	1	6.203	**0.013**	0.017	0.174	-0.003	0.165
Solo howling°	0.142	0.040	1	11.836	**0.001**	0.064	0.222	0.107	0.175
Whining°	0.025	0.040	1	0.387	0.534	-0.054	0.104	-0.019	0.053
Sex§	-0.169	0.147	1	1.233	0.267	-0.451	0.120	-0.259	-0.044
Locomotion°	0.011	0.047	1	0.060	0.806	-0.081	0.098	-0.007	0.037
Feeding status¶	-0.131	0.105	1	1.465	0.226	-0.324	0.079	-0.245	-0.084
Reproductive phase#	0.252	0.123	1	3.677	0.055	-0.013	0.503	0.121	0.344
Species†: Territorial behaviour°	0.170	0.081	1	4.322	**0.038**	0.007	0.325	0.096	0.580

Statistically significant results (P ≤ 0.05) appear in bold. SE, standard error. Df, degrees of freedom. CI, confidence interval. * Not shown because of limited interpretation only.
†, ‡, §, ¶, # χ 2 and P values refer to comparison with the test predictors' reference levels:
† Reference level 'dog'.
§ Reference level 'female'.
¶ Reference level 'not fed/fasted'.
# Reference level 'anestrus'.
° Co-variate (normalized duration, z-transformed).

Table S11: Full and reduced model outputs for the effects of species and condition on the proportions of solo howling. Related to Fig. 6 and STAR Methods.

Full model<br>Predictor	Estimate	SE	Df	χ 2	P
Intercept	-4.533	0.226	NA*	NA*	NA*
Species†	0.025	0.281	NA*	NA*	NA*
Separation condition‡	0.127	0.266	NA*	NA*	NA*
Sex§	0.175	0.159	1	1.198	0.274
Species†: Separation condition‡	0.580	0.355	1	2.696	0.101

Reduced model<br>Predictor	Estimate	SE	Df	χ 2	P	Lower CI	Upper CI	Stability min	Stability max
(Intercept)	-4.711	0.216	NA*	NA*	NA*	-5.146	-4.344	-4.815	-4.670
Species†	0.393	0.168	1	5.539	**0.019**	0.040	0.747	-0.037	-0.002
Separation condition‡	0.464	0.173	1	7.466	**0.006**	0.120	0.831	0.613	0.704
Sex§	0.174	0.160	1	1.166	0.280	-0.144	0.496	-0.145	-0.026

Statistically significant results ($P \leq 0.05$) appear in bold. SE, standard error. Df, degrees of freedom. CI, confidence interval. * Not shown because of limited interpretation only.
†, ‡, § χ^2 and P values refer to comparison with the test predictors' reference levels:
† Reference level 'dog'.
‡ Reference level 'control condition'.
§ Reference level 'female'.

Table S12: Full model output for the effects of species and condition on the proportions of whining. Related to STAR Methods.

Full model<br>Predictor	Estimate	SE	Df	χ 2	P	Lower CI	Upper CI	Stability min	Stability max
Intercept	-5.226	0.359	NA*	NA*	NA*	-5.933	-4.619	-5.359	-5.024
Species†	0.406	0.410	NA*	NA*	NA*	-0.389	1.160	0.288	0.495
Separation condition‡	2.026	0.232	NA*	NA*	NA*	1.647	2.453	1.580	2.463
Sex§	0.037	0.279	1	0.018	0.893	-0.467	0.535	-0.141	0.314
Species†: Separation condition‡	-1.483	0.306	1	18.941	**0.000**	-2.083	-0.921	-1.904	-1.052

Statistically significant results ($P \leq 0.05$) appear in bold. SE, standard error. Df, degrees of freedom. CI, confidence interval. * Not shown because of limited interpretation only.
†, ‡, § χ^2 and P values refer to comparison with the test predictors' reference levels:
† Reference level 'dog'.
‡ Reference level 'control condition'.
§ Reference level 'female'.

Table S13: Full model output for the effects of species and condition on the proportions of locomotion. Related to STAR Methods.

Full model<br>Predictor	Estimate	SE	Df	χ 2	P	Lower CI	Upper CI	Stability min	Stability max
Intercept	-2.170	0.216	NA*	NA*	NA*	-2.598	-1.790	-2.458	-1.948
Species†	0.173	0.290	NA*	NA*	NA*	-0.402	0.773	-0.055	0.479
Induced chorus howling‡	0.278	0.283	NA*	NA*	NA*	-0.216	0.840	0.184	0.402
Separation condition‡	0.768	0.266	NA*	NA*	NA*	0.262	1.275	0.613	1.018
Territorial condition‡	2.315	0.254	NA*	NA*	NA*	1.828	2.811	2.002	2.710
Sex§	0.016	0.115	1	0.019	0.892	-0.217	0.230	-0.043	0.065
Species†: Induced chorus howling‡	-0.134	0.384	3	15.048	**0.002**	-0.878	0.633	-0.293	0.060
Species†: Separation condition‡	0.646	0.356	NA*	NA*	NA*	-0.062	1.377	0.367	0.825
Species†: Territorial condition‡	-0.528	0.370	NA*	NA*	NA*	-1.225	0.215	-0.955	-0.215

Statistically significant results (P ≤ 0.05) appear in bold. SE, standard error. Df, degrees of freedom.
CI, confidence interval. * Not shown because of limited interpretation only.
†, ‡, § χ^2 and P values refer to comparison with the test predictors' reference levels:
† Reference level 'dog'.
‡ Reference level 'control condition'.
§ Reference level 'female'.

Table S14: Full model output for the effect of species and urinary OTM on urinary GCM concentrations in the control condition (baseline samples). Related to Fig. 7 and STAR Methods.

Full model<br>Predictor	Estimate	SE	Df	χ 2	P	Lower CI	Upper CI	Stability min	Stability max
Intercept	1.665	0.225	NA*	NA*	NA*	1.200	2.134	1.340	1.980
Species†	-1.264	0.326	NA*	NA*	NA*	-1.928	-0.656	-1.579	-0.938
OT pg/ml SG°	0.000	0.001	NA*	NA*	NA*	-0.001	0.001	-0.001	0.001
Species†: OT pg/ml SG°	0.003	0.001	1	4.579	**0.032**	0.000	0.006	0.002	0.004

Statistically significant results (P ≤ 0.05) appear in bold. SE, standard error. Df, degrees of freedom.
CI, confidence interval. * Not shown because of limited interpretation only.
† χ^2 and P values refer to comparison with the test predictors' reference level:
† Reference level 'dog'.
° Co-variate.

Table S15: Full and reduced model outputs for the effect of species and urinary OTM on urinary GCM concentrations in the induced chorus howling condition. Related to Fig. 8 and STAR Methods.

**Full model**<br>**Predictor**	**Estimate**	**SE**	**Df**	**χ 2**	**P**
Intercept	1.406	0.238	NA*	NA*	NA*
Species†	-0.430	0.328	NA*	NA*	NA*
OT pg/ml SG°	0.001	0.001	NA*	NA*	NA*
Species†: OT pg/ml SG°	0.000	0.001	1	0.138	0.711

**Reduced model**<br>**Predictor**	**Estimate**	**SE**	**Df**	**χ 2**	**P**	**Lower CI**	**Upper CI**	**Stability min**	**Stability max**
(Intercept)	1.449	0.209	NA*	NA*	NA*	1.002	1.857	1.297	1.508
Species†	-0.532	0.183	1	6.556	**0.010**	-0.926	-0.137	-0.633	-0.336
OT pg/ml SG°	0.001	0.001	1	3.705	**0.054**	0.000	0.002	0.001	0.002

Statistically significant results (P ≤ 0.05) appear in bold. SE, standard error. Df, degrees of freedom. CI, confidence interval. * Not shown because of limited interpretation only.
† χ 2 and P values refer to comparison with the test predictors' reference level:
† Reference level 'dog'.
° Co-variate.
